# Growth Month-Associated Variation in Volatile Profiles, Anti-Glycation Capacity, and Antioxidant Activity of *Cyclocarya paliurus* Leaves: A Pilot Study

**DOI:** 10.3390/foods15122183

**Published:** 2026-06-17

**Authors:** Yanmeng Fu, Qiyue Shao, Liang Chen, Tianxiao Zhang, Jingyi Zhao, Wenhui Zhou, Bin Long, Dai Lu, Wei Wang, Xing Tian

**Affiliations:** 1 TCM and Ethnomedicine Innovation & Development International Laboratory, Innovative Material Medical Research Institute, School of Pharmacy, Hunan University of Chinese Medicine, Changsha 410208, China; 17779977736@163.com (Y.F.); sqiqi7777777@163.com (Q.S.);; 2 School of Food Science and Technology, Jiangnan University, 1800 Lihu Road, Wuxi 214122, China; 3 Department of Food and Drug Engineering, School of Pharmacy, Hunan University of Chinese Medicine, Changsha 410208, China; 4 Hunan Engineering and Technology Research Center for Health Products and Life Science, Changsha 410208, China

**Keywords:** *Cyclocarya paliurus* leaves, volatile compounds, anti-glycation capacity, antioxidant activity, Solid Phase Micro-Extraction Gas Chromatography-Mass Spectrometry (SPME-GC-MS)

## Abstract

*Cyclocarya paliurus* leaves, commonly consumed as “sweet tea”, remain underutilized after spring harvest. This pilot study investigated harvest month-associated changes in bioactivity-related properties and volatile profiles of mature leaves collected from May to September (Q5–Q9) at one site in 2024. Aqueous extracts were analyzed for TPC, TFC, TP, TSC, DPPH and ABTS•+ radical scavenging activities, and inhibition of advanced glycation end-products (AGEs) formation in glucose/fructose-BSA models, with aminoguanidine as a positive reference. Dried leaf powders were profiled by SPME-GC-MS for tentative VOC annotation. TPC, TFC, TP, and TSC ranged from 28.04 to 28.87, 15.42 to 40.22, 2.14 to 2.51, and 23.15 to 25.30 mg/g, respectively. Q9 showed the strongest radical scavenging response, with the lowest DPPH and ABTS•+ IC50 values (0.119 and 0.131 mg/mL), while Q6 also exhibited relatively strong activity. Furthermore, Q6 and Q9 exhibited superior advanced glycation end-product (AGE) inhibitory responses, with Q9 being particularly effective in the fructose-BSA model. VOC profiles varied markedly by month, shifting from alkene/terpene predominance in Q5–Q6 to alcohol enrichment in Q7 and renewed alkene/terpene predominance in Q9. Integrated heatmap and Pearson correlation analyses identified clear temporal co-variations and pairwise associations between distinct VOC classes and bioactivity indices. Collectively, these results provide preliminary, site- and year-specific evidence that harvest month is associated with changes in the bioactivity-related properties and aroma-related phytochemical profiles of mature *C. paliurus* leaves, offering a cautious reference for harvest-stage-oriented utilization.

## 1. Introduction

*Cyclocarya paliurus* (Batalin) Iljinskaja (*C. paliurus*), also known as the sweet tea tree, is a deciduous tree belonging to the genus *Cyclocarya* of the family Juglandaceae. It is one of the endemic plant resources in China, mainly distributed in the middle and lower reaches of the Yangtze River [1]. Owing to their natural sweetness, its leaves have long been consumed as a traditional “sweet tea” and used in folk practice [2]. In China, *C. paliurus* leaves were officially approved as a novel food ingredient on 30 October 2013, and permitted for infusion use under the relevant national regulatory framework [3]. Currently, functional tea products derived from the immature leaves of *C. paliurus* have achieved large-scale industrialization. Despite the established industry, current production primarily focuses on the tender leaves harvested in April. Consequently, a substantial volume of mature leaves from subsequent growth stages remains underutilized, leading to a significant waste of potential resources. Previous studies have shown that mature *C. paliurus* leaves possess potential health-promoting properties, including hypoglycemic, hypolipidemic, and anti-aging effects, suggesting promising prospects for their high-value utilization [4]. Therefore, with increasing interest in the edible and health-promoting value of *C. paliurus*, its chemical composition and biological activities have gradually become research hotspots.

In recent years, increasing attention has been paid to *C. paliurus* in the context of functional foods and natural plant resources. A growing body of evidence has shown that its leaves contain multiple bioactive constituents, including flavonoids and their glycosides, polyphenols, phenolic acids, triterpenoids, and polysaccharides [5,6,7]. These compounds are considered important contributors to the antioxidant and anti-glycation potential of the leaves. Both activities are of particular interest because oxidative stress and non-enzymatic glycation are closely involved in aging and the development of chronic metabolic disorders. Antioxidants can reduce oxidative injury by scavenging reactive oxygen species, inhibiting lipid peroxidation, and protecting biological macromolecules [8,9], whereas anti-glycation agents may suppress the formation of advanced glycation end-products (AGEs) and thereby mitigate glycation-associated damage [10,11]. Accordingly, antioxidant and anti-glycation evaluations are important for assessing the functional potential of *C. paliurus* leaves.

Substantial evidence has confirmed the antioxidant capacity of *C. paliurus* leaves. In vitro assays, including 2,2-diphenyl-1-picrylhydrazyl (DPPH), ABTS, FRAP, and hydroxyl and superoxide anion radical scavenging models, have demonstrated notable radical-scavenging activity and total antioxidant capacity [12,13]. Further animal experiments have demonstrated that *C. paliurus* leaves can significantly enhance the activities of endogenous antioxidant enzymes, including superoxide dismutase (SOD), catalase (CAT), and glutathione peroxidase (GSH-Px), while reducing lipid peroxidation levels, thereby exerting protective effects against oxidative stress-related damage in the liver, pancreas, and kidneys [14,15]. Regarding anti-glycation and hypoglycemic effects, component isolation and in vivo studies have shown that the flavonoid and polysaccharide fractions are the main contributors to the antihyperglycemic activity of *C. paliurus* leaves [16]. Studies based on type 2 diabetic animal models induced by streptozotocin (STZ) or by a high-fat diet combined with low-dose STZ further revealed that *C. paliurus* leaf extracts significantly reduce fasting blood glucose levels, improve glucose tolerance and insulin sensitivity, and exert hypoglycemic and pancreatic protective effects by inhibiting pancreatic β-cell apoptosis and maintaining islet structural integrity [17,18,19]. Additional studies have also suggested potential protective effects against diabetic nephropathy and related complications, partly through aldose reductase inhibition and attenuation of oxidative stress [20].

In parallel with these functional studies, the volatile constituents of *C. paliurus* leaves have also attracted increasing interest. Solid-phase microextraction coupled with gas chromatography–mass spectrometry (SPME-GC-MS) has been widely used to characterize their volatile composition, and previous studies have identified alcohols, aldehydes, ketones, esters, olefins, and terpenoids as major contributors to the characteristic aroma of *C. paliurus* tea [21,22,23]. From the dual perspectives of leaf ontogeny and metabolic regulation, existing literature demonstrates that the concentrations of flavonoids, phenolic acids, alkaloids, and terpenoids in *C. paliurus* leaves do not remain static during growth. Instead, these secondary metabolites undergo dynamic flux, facilitated by a series of continuous biochemical transformations, including degradation, methylation, glycosylation, redox reactions, and isomerization [24]. Consequently, significant differences in metabolic intensity and pathways occur at different growth stages, leading to stage-dependent changes in chemical composition and biological activities. This dynamic variation not only modulates the accumulation and biotransformation of non-volatile bioactive constituents (such as flavonoids, polyphenols, and glycosides), but may also govern the biosynthesis and emission of volatile compounds via precursor conversion and metabolically coupled pathways. Therefore, changes in volatile profiles may not only reflect aroma variation, but may also be associated with broader metabolic shifts related to antioxidant and anti-glycation activities.

Collectively, contemporary research on *C. paliurus* leaves has evolved from the fundamental identification of chemical constituents to comprehensive functional characterizations, including antioxidant activity, hypoglycemic effects, and metabolic regulation. Concurrent advancements have also been documented in the profiling of volatile organic compounds (VOCs). However, existing studies have predominantly focused on comparing chemical profiles across different geographical origins to address spatial diversity [12,22], or on assessing specific bioactivities at a single harvest stage or partial developmental windows to establish baseline functional values [25,26]. While these investigations have provided fundamental insights, systematic investigations concerning the coordinated dynamic fluctuation in volatile profiles, antioxidant capacity, and anti-glycation potential throughout the entire growth cycle remain significantly underrepresented. To date, no systematic seasonal dynamic profiling integrating volatile variation with antioxidant and anti-glycation activities in *C. paliurus* leaves has been clearly reported.

Therefore, it is necessary to systematically investigate the dynamic changes in volatile composition and health-related activities of *C. paliurus* leaves across different harvest months, particularly in underutilized mature leaves. In this study, we aimed to characterize month-dependent variations in volatile profiles, antioxidant capacity, and anti-glycation activity of *C. paliurus* leaves harvested from May to September, and to visualize coordinated temporal patterns among these parameters across harvest months.

## 2. Materials and Methods

### 2.1. Reagents and Chemicals

*C. paliurus* leaf samples collected at different growth stages were obtained from Hunan Yueling Junshan Agroforestry Technology Co., Ltd. (Shimen, Hunan, China). Folin–Ciocalteu phenol reagent was purchased from Hefei Bomei Biotechnology Co., Ltd. (Hefei, Anhui, China). Anhydrous ethanol was supplied by Anhui Ante Food Co., Ltd. (Suzhou, Anhui, China), while phenol was obtained from Guangdong Guanghua Technology Co., Ltd. (Guangzhou, Guangdong, China). Sulfuric acid and perchloric acid were purchased from Hunan Huihong Reagent Co., Ltd. (Changsha, Hunan, China). The following guaranteed reagents were provided by Chengdu Lemeitian Pharmaceutical Technology Co., Ltd. (Chengdu, China): gallic acid, rutin solution, glucose, fructose, methylglyoxal (MGO; 40% aqueous solution), bovine serum albumin (BSA), o-phenylenediamine (OPD), hydrochloric acid, phosphate-buffered saline (PBS), 2,2-diphenyl-1-picrylhydrazyl (DPPH), β-mercaptoethanol, NaHCO_3_, Tris and oleanolic acid. Aminoguanidine hydrochloride (AG; Sigma-Aldrich, St. Louis, MO, USA) was used as the positive reference inhibitor in the anti-glycation assays. An ABTS radical scavenging assay kit (ADS-F-AB001, spectrophotometric method, AIDISHENG, Nanjing, Jiangsu, China) was used for the complementary evaluation of antioxidant activity. Anhydrous sodium carbonate, sodium nitrite, aluminum nitrate, vanillin, and glacial acetic acid were purchased from Sinopharm Chemical Reagent Co., Ltd. (Shanghai, China).

### 2.2. Collection and Preparation of C. paliurus Leaf Samples

*C. paliurus* leaf samples were collected from Huping Mountain, Shimen County, Hunan Province, P.R. China (110.773665° E, 29.94036696° N). To investigate seasonal changes in leaf constituents, fresh, fully expanded, mature leaves were collected at approximately monthly intervals during the 2024 growing season (3 May, 1 June, 3 July, 6 August, and 1 September, corresponding to Q5–Q9). To improve sampling representativeness, the plantation was divided into three sampling blocks, with approximately 20 *C. paliurus* trees in each block. At each sampling time point, approximately 200 g of fresh, fully expanded, mature leaves were randomly collected from multiple trees within each block and from different canopy positions. The samples from the three blocks were then oven-dried at 60 °C to constant weight, ground into fine powder, and stored at −20 °C until analysis. Thus, the three sampling blocks served as three independent biological replicates for each sampling month.

To account for potential environmental influences on metabolite variation, monthly meteorological data during the sampling period (May–September 2024), including rainfall, rainy days, and maximum, mean, and minimum temperatures, were obtained from the WorldWeatherOnline website (https://www.worldweatheronline.com, accessed on 6 March 2026) and are summarized in Table S1.

### 2.3. Preparation of Aqueous Extracts from C. paliurus Leaves

Aqueous extraction was used because *C. paliurus* leaves are traditionally consumed as an infusion, making water extracts more relevant to their practical dietary and functional applications. Leaf powders from each biological replicate were extracted independently. For each leaf powder sample, 6.0 ± 0.01 g was mixed with 120 mL of distilled water in a 250 mL round-bottom flask and extracted at 90 °C for 40 min in a water bath. The extract was then centrifuged at 4500 rpm for 5 min at 4 °C, and the supernatant was filtered three times through three layers of gauze. The combined filtrates were concentrated under reduced pressure (75 kPa) at 65 °C and then freeze-dried (Xinzhi Freeze-drying Equipment Co., Ltd., Ningbo, China). The resulting lyophilized aqueous extract powder was sealed and stored at −20 °C until analysis. The resulting lyophilized aqueous extract powder was weighed, and the extraction yield was calculated as follows: Extraction yield (%) = (weight of lyophilized extract powder/weight of dry leaf powder) × 100. Under the investigated conditions, the extraction yields of *C. paliurus* leaf aqueous extracts ranged from 17.8% to 20.1% among different harvest months.

### 2.4. Determination of Total Phenolic Content (TPC), Total Flavonoid Content (TFC), Total Polysaccharide Content (TP), and Total Saponin Content (TSC)

The lyophilized aqueous extract powder of *C. paliurus* leaves was used for the determination of total phenolic content (TPC), total flavonoid content (TFC), total polysaccharide content (TP), and total saponin content (TSC). All constituent contents were expressed on a dry weight basis of the lyophilized aqueous extract powder.

The total phenolic content (TPC) of the lyophilized aqueous extract powder of *C. paliurus* leaves was determined using the Folin–Ciocalteu colorimetric method, as previously described by Xiao et al. [27]. Quantification was performed using a gallic acid calibration curve, and results were expressed as milligrams of gallic acid equivalents per gram of dry weight (mg GAE/g DW). The total flavonoid content (TFC) was measured by the aluminum nitrate colorimetric method according to Yang et al. [28]. A rutin standard curve was employed for calibration, and the results were reported as milligrams of rutin equivalents per gram of dry weight (mg RE/g DW). The total polysaccharide content (TP) was determined using the phenol–sulfuric acid method [29], with glucose used as the reference standard. Data were expressed as milligrams of glucose equivalents per gram of dry weight (mg GE/g DW). The total saponin content (TSC) was quantified following the spectrophotometric method described by de Aguiar et al. [30], which involves acid hydrolysis to release triterpene aglycones, followed by vanillin-based color development. Quantification was based on an oleanolic acid standard curve, and results were expressed as milligrams of oleanolic acid equivalents per gram of dry weight (mg OAE/g DW).

### 2.5. Antioxidant Evaluation of C. paliurus Leaf Extracts Using DPPH and ABTS•+ Radical Scavenging Assays

A methanolic solution of 2,2-diphenyl-1-picrylhydrazyl (DPPH) (0.315 mM) was freshly prepared prior to analysis. The lyophilized aqueous extract powder of *C. paliurus* leaves was dissolved and serially diluted (20–200-fold) to obtain final extract concentrations of 0.2, 0.5, 1.0, 2.0, 3.5, and 5.0 mg/mL in the reaction mixture. For the assay, 20 μL of each diluted sample was mixed with 200 μL of the DPPH solution in disposable optical polystyrene 96-well plates [31]. The reaction mixture was incubated at room temperature in the dark for 30 min, after which the absorbance was measured at 517 nm using a microplate spectrophotometer. All measurements were performed in triplicate. Vitamin C (VC) was used as a standard positive control and tested under the same conditions. The free radical scavenging activity was inversely proportional to the absorbance value. The scavenging activity (%) was calculated using the following equation [32]:

$$Inhibition(\%)=\frac{{ABS}_{control}-({ABS}_{sample}-{ABS}_{blank})}{{ABS}_{control}}\times 100$$

${ABS}_{control}$: Absorbance of the 220 μL DPPH methanolic solution without sample;

${ABS}_{sample}$: Absorbance of the reaction mixture containing 20 μL of sample solution at various concentrations and 200 μL of DPPH methanolic solution;

${ABS}_{blank}$: Absorbance of the 220 μL sample solutions at various concentrations without DPPH.

The IC_50_ value, defined as the concentration required to scavenge 50% of DPPH radicals, was calculated by nonlinear regression analysis using Origin 2021.

The ABTS•+ radical scavenging activity of the lyophilized aqueous extracts was further determined as a complementary antioxidant evaluation using a commercial ABTS radical scavenging assay kit (ADS-F-AB001, AIDISHENG, Nanjing, Jiangsu, China). Briefly, reagent I and reagent II were dissolved according to the manufacturer’s instructions, mixed at a ratio of 1:1, and allowed to react in the dark for 12 h to generate the ABTS•+ stock solution. Prior to measurement, the stock solution was diluted 25-fold with absolute ethanol to prepare the ABTS•+ working solution, with the absorbance of the reagent blank at 734 nm maintained within the recommended range of 1.0 ± 0.2.

The extract solutions were prepared at concentrations of 0.05, 0.10, 0.15, 0.20, 0.30, and 0.60 mg/mL. For each determination, 50 μL of sample solution was mixed with 950 μL of ABTS•+ working solution. The sample control consisted of 50 μL of sample solution and 950 μL of absolute ethanol, while the reagent blank consisted of 50 μL of absolute ethanol and 950 μL of ABTS•+ working solution. The mixtures were incubated at room temperature (approximately 25 °C) in the dark for 6 min, and absorbance was measured at 734 nm. Vitamin C (VC) was used as the positive control and tested under the same conditions. All measurements were performed in triplicate.

The ABTS•+ radical scavenging activity was calculated using the following equation:

$$Inhibition(\%)=[1-\frac{\left({ABS}_{sample}-{ABS}_{control}\right)}{{ABS}_{blank}}]\times 100$$

where ${ABS}_{sample}$ is the absorbance of the reaction mixture containing sample solution and ABTS•+ working solution,

${ABS}_{control}$ is the absorbance of the sample control without ABTS•+ working solution, and ${ABS}_{blank}$ is the absorbance of the reagent blank without sample solution.

The IC_50_ value, defined as the sample concentration required to scavenge 50% of ABTS•+ radicals, was calculated by nonlinear regression analysis using Origin 2021.

### 2.6. Anti-Glycation Evaluation of C. paliurus Leaf Extracts in BSA-Based Glycation Models

To evaluate the anti-glycation activity of *C. paliurus* leaf extracts, two in vitro glycation models, namely glucose–BSA (Glu–BSA) and fructose–BSA (Fru–BSA), were established. For the Glu–BSA model, bovine serum albumin (BSA; 50 mg mL^−1^) and *d*-glucose (0.8 M) were dissolved in 100 mL of phosphate-buffered saline (PBS), corresponding to 5 g BSA and 14.4 g glucose. The Fru–BSA model was prepared in an analogous manner by dissolving 5 g BSA and 45.05 g fructose in 100 mL PBS to obtain final concentrations of 50 mg mL^−1^ BSA and 2.5 M fructose. For each glycation system, 1 mL of the reaction mixture was combined with 50 μL of the test solution. The test solution was prepared by re-dissolving the lyophilized aqueous extract powder of *C. paliurus* leaves in methanol at 50 mg/mL. Methanol was used as a uniform reconstitution solvent so that all samples were tested under the same conditions. The final extract concentration in the reaction system was 2.38 mg/mL. Aminoguanidine hydrochloride (AG; Sigma, USA) was included as a positive reference inhibitor according to a previously reported method with minor modifications [33]. AG was dissolved in methanol at the same mass concentration as the leaf extracts (50 mg/mL). For the AG group, 50 μL of AG solution was added to 1 mL of the corresponding glycation reaction mixture, resulting in a final AG concentration of 2.38 mg/mL. The anti-glycation assay in the present study was conducted at a single fixed concentration and was intended to compare month-associated differences among leaf extracts under the investigated conditions, rather than to provide a complete dose–response potency assessment or a direct equivalence comparison with AG. The corresponding negative control received 50 μL of methanol instead of the leaf extract or AG solution. Sodium azide (NaN_3_) was added to all reaction mixtures at a final concentration of 0.2 g L^−1^ to prevent microbial contamination. The reaction mixtures were incubated at 37 °C for 4 weeks. The formation of advanced glycation end-products (AGEs) was monitored weekly by measuring fluorescence intensity. At each time point, 200 μL of the reaction mixture was transferred to a black 96-well microplate, and fluorescence was recorded using a Victor X4 Multilabel Plate Reader (PerkinElmer, Waltham, MA, USA) at excitation and emission wavelengths of 330 nm and 410 nm, respectively. Relative AGE_s_ fluorescence was calculated according to Equation (1), and the corresponding AGE inhibition rate was calculated according to Equation (2) [34].

(1) $$\mathrm{Relative}\;\mathrm{AGE}\;\mathrm{fluorescence}=\frac{{\mathrm{Trt}}_{n}}{{\mathrm{Ctl}}_{0}}$$

(2) $$\mathrm{AGE}\;\mathrm{inhibition}\left(\%\right)=\left(1-\frac{{\mathrm{Trt}}_{n}}{{\mathrm{Ctl}}_{n}}\right)\times 100$$

Trt_n_: fluorescence intensity of the glycation system containing *C. paliurus* leaf extract or AG at week n;

Ctl_0_: fluorescence intensity of the corresponding control solution at week 0;

Ctl_n_: fluorescence intensity of the corresponding control solution at week n.

### 2.7. Solid Phase Micro-Extraction Gas Chromatography–Mass Spectrometry (SPME-GC-MS) Analysis

For volatile analysis, the oven-dried leaf powders, prepared as described in Section 2.2, were used. Approximately 0.50 ± 0.01 g of the powder was accurately weighed into a 15 mL headspace vial and equilibrated in a thermostatic water bath at 60 °C for 10 min. A preconditioned solid-phase microextraction (SPME) fiber, conditioned at 250 °C for 30 min, was then exposed to the headspace for extraction, and adsorption was allowed to proceed for 45 min. Immediately after extraction, the SPME fiber was inserted into the GC injection port and thermally desorbed at 250 °C for 5 min for subsequent GC-MS analysis.

GC-MS conditions: Volatile compounds were analyzed using an HP-5MS capillary column (30 m × 0.25 mm × 0.25 μm; Agilent, Santa Clara, CA, USA). Helium (99.999%) was used as the carrier gas at a constant flow rate of 1.0 mL/min. The oven temperature program was as follows: the initial temperature was 40 °C and held for 3 min; it was increased to 90 °C at 2 °C/min, then raised to 150 °C at 3 °C/min, further increased to 180 °C at 5 °C/min, and finally raised to 230 °C at 15 °C/min and held for 5 min.

MS conditions: Mass spectrometry was performed in electron impact (EI) mode at 70 eV. The ion source temperature was set at 230 °C. Mass spectra were acquired in full-scan mode over the range of *m*/*z* 35–400.

Compound identification and semi-quantification: Volatile compounds were tentatively identified by comparing their mass spectra with those in the NIST 17 library and were further screened by retention index (RI) comparison. RI values were calculated using a homologous series of n-alkanes analyzed under the same chromatographic conditions, and the calculated values were compared with reference RI values reported for comparable low-polarity columns. Compounds with an RI difference greater than 30 were excluded. For transparency, retention times (RT), calculated RI values, and reference RI values are provided in Supplementary Table S2. The relative contents of volatile compounds were expressed as relative peak-area percentages based on peak-area normalization. Therefore, the reported VOC annotations should be interpreted as tentative identifications.

### 2.8. Statistical Analysis

Data are presented as mean ± SD of three biological replicates per harvest month (*n* = 3). Differences among harvest months were analyzed by one-way ANOVA followed by Duncan’s multiple range test using SPSS 25.0 (IBM, Armonk, NY, USA), with significance at *p* < 0.05. IC50 values for DPPH and ABTS•+ radical scavenging assays were estimated by nonlinear regression in Origin 2021 (OriginLab, Northampton, MA, USA). Hierarchical clustering and the integrated temporal heatmap were generated in TBtools (version 2.483, South China Agricultural University, China) based on normalized data. Pairwise Pearson correlation analysis was conducted as an exploratory analysis in SPSS 25.0 using 15 matched biological replicate-level measurements to examine co-variation among selected VOC classes, bioactive constituent indices (TPC, TFC, TP, TSC), and bioactivity indices, including DPPH IC_50_, ABTS•+ IC_50_, and week-4 AGE inhibition rates in the Glu–BSA and Fru–BSA models, without implying causal relationships. The correlation matrix was visualized as a triangular heatmap in Origin 2021. Original IC_50_ values were retained without transformation; lower IC_50_ indicates stronger radical scavenging activity. The resulting correlations were considered exploratory pairwise associations and should not be interpreted as causal relationships. Multivariate analyses (PCA, OPLS-DA) were performed using SIMCA 14.1 (Umetrics, Umeå, Sweden). Other figures were prepared using Origin 2021.

## 3. Results

### 3.1. Changes in TPC, TFC, TP, and TSC of C. paliurus Leaves in Different Growth Periods

**Figure 1:**
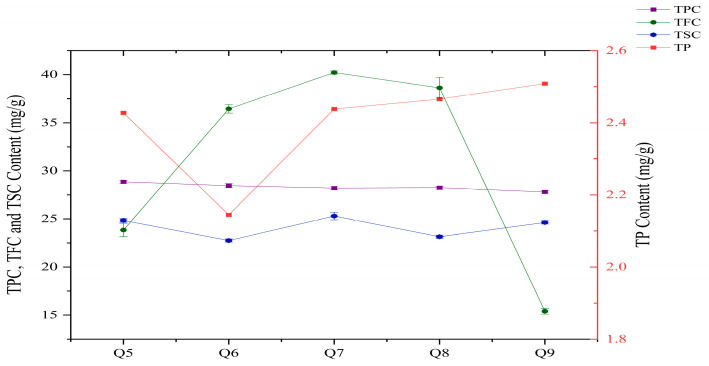
Changes in the TPC, TFC, TSC, and TP of *C. paliurus* leaves in five different months. Note: TPC (Total Phenolic Content), TFC (Total Flavonoid Content), TP (Total Polysaccharide Content), and TSC (Total Saponin Content). Q5–Q9 denote *C. paliurus* leaf samples collected from May to September. Data are expressed as mean ± SD (n = 3). Error bars are shown for all data points.

The extraction yields of aqueous extracts from *C. paliurus* leaves showed moderate month-associated variation, ranging from 17.8% to 20.1% under the investigated conditions (Table S1). The contents of total phenolic content (TPC), total flavonoid content (TFC), total saponin content (TSC), and total polysaccharide content (TP) in *C. paliurus* leaves exhibited distinct growth stage-dependent variation patterns (Figure 1). Among these components, TPC remained relatively stable from Q5 to Q9, showing only minor fluctuations, indicating that phenolic accumulation was largely maintained throughout the main growing season with minimal dilution effects from leaf expansion. In contrast, TFC displayed pronounced temporal variation, increasing markedly from Q5 to reach a maximum at Q7, remaining at a relatively high level at Q8, and then decreasing sharply at Q9. This trend suggests that flavonoid biosynthesis is strongly influenced by developmental stage and seasonal environmental conditions, such as increased irradiance and temperature during active growth. Similar mid-summer peaks in flavonoid accumulation have been reported for *C. paliurus* leaves and are supported by recent metabolomics studies showing substantial seasonal variation in secondary metabolites [1,35,36,37]. Compared with TFC, TSC exhibited relatively limited variation across the growth stages, with only a modest increase at Q7, implying a more tightly regulated biosynthesis or slower metabolic turnover of saponins. TP showed a distinct V-shaped pattern, decreasing from Q5 to a minimum at Q6 and subsequently increasing toward Q9. The early decrease may be associated with rapid leaf expansion–induced dilution, whereas the later increase likely reflects enhanced polysaccharide accumulation or a concentration effect at later developmental stages. In general, TFC and TP showed greater seasonal variability than TPC and TSC, indicating that flavonoids and polysaccharides are the most growth stage-responsive constituents in *C. paliurus* leaves and may therefore play key roles in determining their functional and nutritional properties.

### 3.2. Changes in Antioxidant Activity

**Figure 2:**
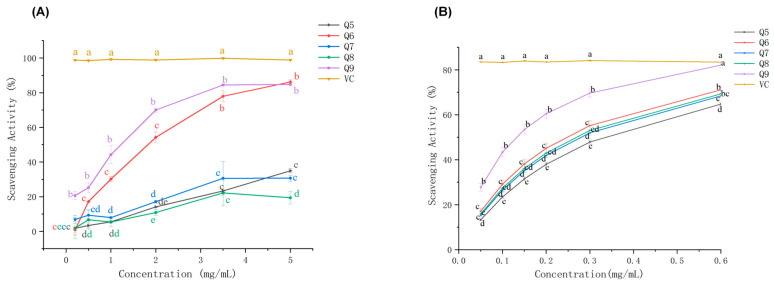
Concentration-dependent radical scavenging activities of *C. paliurus* leaf extracts collected in different months. Notes: (**A**) DPPH radical scavenging activity and (**B**) ABTS radical cation (ABTS•+) scavenging activity. VC refers to vitamin C used as the positive control. Q5–Q9 denote *C. paliurus* leaf samples collected from May to September. Data are presented as mean ± standard deviation (SD, n = 3). Within each assay, different lowercase letters at the same tested concentration indicate statistically significant differences among samples, including VC, according to one-way ANOVA followed by Duncan’s multiple range test (*p* < 0.05).

The DPPH radical scavenging activity of *C. paliurus* leaf extracts increased in a concentration-dependent manner for all sampling months (Figure 2A), showing typical dose-response behavior. However, marked differences in radical scavenging responses were observed among growth periods. Extracts from Q9 and Q6 exhibited relatively stronger scavenging activity than those from the other months across the tested concentration range. At 5 mg/mL, the scavenging rates of Q6 and Q9 exceeded 80%, approaching that of the positive control (vitamin C), whereas Q5, Q7, and Q8 showed relatively weaker activities. These differences were further reflected by the DPPH IC_50_ values (Table 1), with Q9 showing the lowest IC_50_ value (0.119 mg/mL), followed by Q6 (0.222 mg/mL), indicating relatively stronger DPPH radical scavenging responses at the later and mid-growth stages under the investigated conditions.

The complementary ABTS•+ radical scavenging assay further revealed month-associated differences in the antioxidant response of *C. paliurus* leaf extracts (Figure 2B). The ABTS•+ scavenging response of all extracts generally increased with increasing concentration. Among the tested leaf extracts, Q9 exhibited the highest ABTS•+ scavenging response across the concentration range and showed the lowest IC_50_ value among the tested extracts (0.131 ± 0.011 mg/mL; Table 1). Q6 had the second-lowest numerical IC_50_ value of 0.244 ± 0.022 mg/mL, followed by Q8 (0.266 ± 0.022 mg/mL) and Q7 (0.277 ± 0.011 mg/mL), whereas Q5 showed the highest IC_50_ value of 0.327 ± 0.011 mg/mL. Although Q6 showed a numerically lower ABTS•+ IC_50_ value than Q8, the difference between these two months was not statistically significant.

Taken together, the DPPH and ABTS•+ assays consistently indicated that Q9 showed relatively strong radical scavenging responses among the tested harvest months under the investigated conditions. Q6 also exhibited comparatively strong radical scavenging responses, particularly in the DPPH assay. However, the relative ranking of the remaining extracts was not completely identical between the two assays, suggesting that the observed antioxidant response was partly dependent on the radical scavenging system employed.

The month-associated differences in radical scavenging responses may reflect variation in the overall phytochemical composition of the aqueous extracts during leaf development. Recent studies have suggested that flavonoids and water-soluble polysaccharides may contribute to the antioxidant activity of *C. paliurus* leaf extracts [36,37,38]. In the present study, the observed radical scavenging responses may also be associated with these phytochemical constituents; however, the specific active compounds and their underlying mechanisms were not directly investigated. Therefore, the antioxidant results should be interpreted as an overall radical scavenging response associated with the phytochemical composition of the extracts, rather than as evidence of the action of specific compounds or pathways.

### 3.3. Changes in Anti-Glycation Capacity

**Figure 3:**
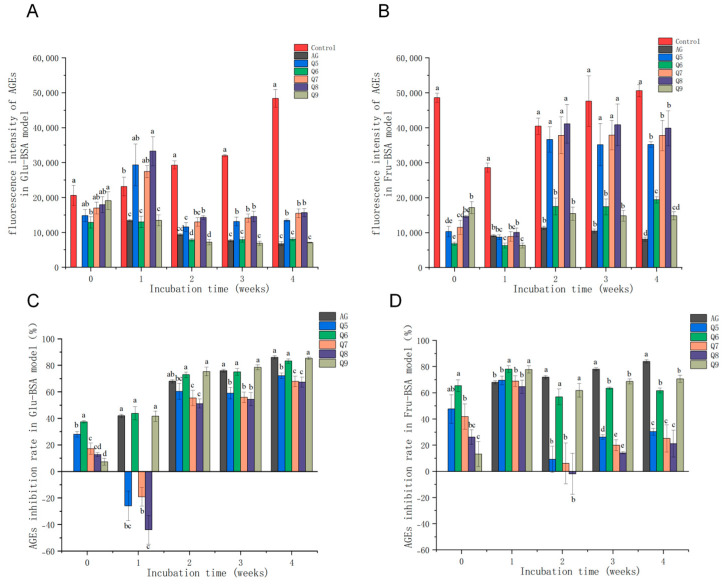
Anti-glycation activity of *C. paliurus* leaf extracts collected in different months, evaluated in glucose–bovine serum albumin (Glu–BSA) and fructose–bovine serum albumin (Fru–BSA) models during 4 weeks of incubation. Note: (**A**) Relative fluorescence intensity of AGEs in the Glu–BSA model; (**B**) relative fluorescence intensity of AGEs in the Fru–BSA model; (**C**) AGE inhibition rate in the Glu–BSA model; and (**D**) AGE inhibition rate in the Fru–BSA model. Aminoguanidine hydrochloride (AG) was included as a positive reference inhibitor. AGEs, advanced glycation end-products; Q5–Q9 denote *C. paliurus* leaf samples collected from May to September. Data are presented as mean ± SD (n = 3). Different lowercase letters above the bars indicate significant differences among the groups shown at the same incubation week within the same model (*p* < 0.05).

The anti-glycation activity of *C. paliurus* leaf extracts was evaluated using both Glu–BSA and Fru–BSA models over prolonged incubation, with aminoguanidine hydrochloride (AG) included as a positive reference inhibitor (Figure 3. In both models, the fluorescence intensity of AGEs in the control groups increased steadily with incubation time, confirming the progressive formation of glycation end-products. In contrast, AG and all leaf extracts reduced AGEs accumulation to varying extents, with differences observed among the tested groups and growth periods. In the Glu–BSA system, extracts from Q6 and Q9 generally exhibited lower AGEs fluorescence intensity than those from the other harvest months, particularly from week 2 onward, indicating relatively stronger AGE inhibitory responses at the tested fixed concentration. A similar trend was observed in the Fru–BSA model, in which Q9 showed the lowest AGEs fluorescence intensity among the leaf extracts during weeks 2–4 at the tested fixed concentration, followed by Q6.

These observations were further supported by the AGE inhibition rates, although the time-course patterns differed between the two glycation systems. In the Glu–BSA model, Q6 and Q9 showed the highest inhibition rates among the leaf extracts at the tested fixed concentration from week 2 onward and exceeded 70% during weeks 3–4. At week 4, Q6 and Q9 showed relatively strong inhibitory responses in the Glu–BSA model under the tested conditions; however, this single fixed-concentration design does not allow direct potency comparison or equivalence assessment with AG. In the Fru–BSA model, Q9 showed a relatively higher AGE inhibitory response among the tested leaf extracts at the tested fixed concentration during weeks 2–4, followed by Q6, whereas Q5, Q7, and Q8 generally showed comparatively weaker responses. At week 4, AG exhibited a significantly higher inhibition rate than all leaf extracts, while Q9 and Q6 showed significantly higher inhibition rates than Q5, Q7, and Q8. These results indicate that the AGE inhibitory responses of *C. paliurus* leaf extracts varied across harvest months under the investigated single fixed-concentration conditions, with Q6 and Q9 showing relatively stronger responses among the tested leaf extracts, while AG provided a positive reference for interpreting the assay results.

This harvest month-dependent difference may be related to developmental changes in the biosynthesis and accumulation of phenolic acids, polysaccharides, and other bioactive constituents, as previously suggested by metabolomic and transcriptomic studies of *C. paliurus* leaves [39,40]. This trend was also broadly consistent with the radical scavenging results obtained from the DPPH and ABTS•+ assays, suggesting temporal co-variation between radical scavenging responses and AGE inhibition under the tested conditions, although direct mechanistic evidence was not obtained in the present study. Previous studies have also reported that polyphenol-rich plant extracts can inhibit AGEs formation and that such effects are associated with their phenolic composition [41]. Since reactive oxygen species, carbonyl intermediates, and specific glycation-related pathways were not directly evaluated in this study, the proposed relationship between radical scavenging responses and AGE inhibition remains speculative and requires further mechanistic investigation.

Moreover, the distinct responses observed between the Glu–BSA and Fru–BSA models suggest that differences in extract composition may be associated with sugar-specific inhibitory patterns. Fructose is known to exhibit higher glycation reactivity than glucose due to its greater proportion of open-chain structures, leading to more rapid and severe AGEs formation. The comparatively stronger inhibitory response of Q9 in the Fru–BSA model may therefore indicate that late-harvest extracts contain constituents potentially more responsive to fructose-mediated glycation under the tested conditions. Furthermore, previous studies have highlighted the bioactivity of *C. paliurus* polysaccharides, including antioxidant and immunomodulatory effects in diabetic rats, suggesting that polysaccharide-related constituents may also contribute to the overall functional properties of the leaves [42]. Notably, AG was included as a positive reference inhibitor to support the interpretation of the anti-glycation responses observed under the tested conditions. Because the leaf extracts were evaluated at a single fixed concentration, the present results should be interpreted as comparative inhibitory responses among harvest months rather than as a dose-response potency assessment. Therefore, the relatively stronger responses observed for Q6 and Q9 indicate month-associated differences under the present assay conditions, without establishing biochemical equivalence to AG.

### 3.4. Analysis of VOCs by SPME-GC-MS

**Figure 4:**
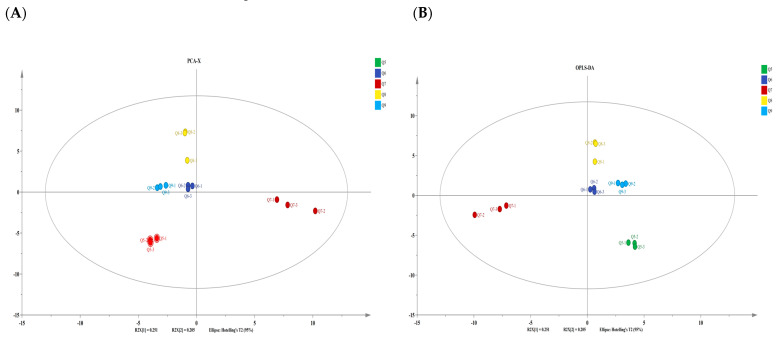
PCA-X and OPLS-DA analyses of VOC profiles in *C. paliurus* leaves collected at different developmental stages (Q5–Q9). Note: (**A**) PCA-X score plot of VOC data from *C. paliurus* leaves collected at different harvest months. (**B**) OPLS-DA score plot showing supervised exploratory variation tendencies among harvest months. For the PCA-X model, R^2^X(cum) = 0.743 and Q^2^(cum) = 0.392. The first two plotted components explained 25.1% and 20.5% of the X-variance, respectively. The 200-times permutation tests yielded R^2^ intercepts of 0.474–0.499 and Q^2^ intercepts of −0.762 to −0.707, suggesting acceptable robustness under the present exploratory conditions. The cross-validation analysis of variance (CV-ANOVA) result was significant (F = 9.99521, *p* = 1.68258 × 10^−8^). The ellipse represents Hotelling’s T^2^ at the 95% confidence level.

**Figure 5:**
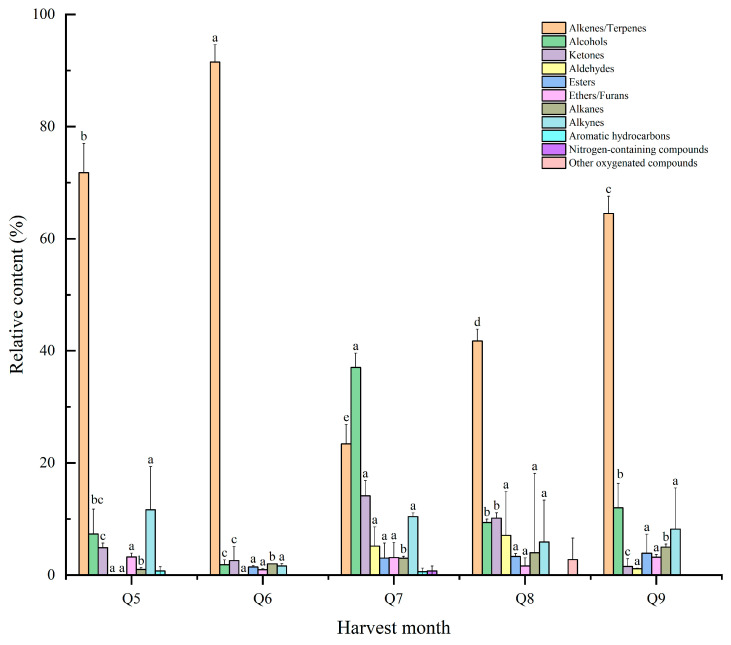
Relative distribution of volatile compound classes in *C. paliurus* leaves harvested at different developmental stages. Note: Volatile compounds were classified into 11 groups: alkenes/terpenes, alcohols, ketones, aldehydes, esters, ethers/furans, alkanes, alkynes, aromatic hydrocarbons, nitrogen-containing compounds, and other oxygenated compounds. Data are expressed as mean ± SD (n = 3) of the relative percentages of the total detected volatile compounds in each harvest month. Different lowercase letters above bars of the same compound class indicate significant differences among harvest months (*p* < 0.05, Duncan’s multiple range test). Q5–Q9 refer to *C. paliurus* leaf samples collected from May to September.

The volatile organic compounds (VOCs) in *C. paliurus* leaves harvested at different developmental stages (Q5–Q9) were tentatively identified and semi-quantified by SPME-GC-MS. The results are summarized at both the chemical-class and individual-compound levels in Figure 4 and Figure 5, Table 2, Table 3 and Table S2. In total, 82 volatile compounds were tentatively annotated across the five harvest months and classified into 11 chemical groups, including alkenes/terpenes, alcohols, ketones, aldehydes, esters, ethers/furans, alkanes, alkynes, aromatic hydrocarbons, nitrogen-containing compounds, and other oxygenated compounds. These compounds exhibited pronounced month-dependent differences in composition and relative abundance, suggesting month-associated variation in VOC profiles during leaf development. Previous chromatographic and mass-spectrometric studies have also demonstrated the value of chemical fingerprinting and volatile profiling for characterizing *C. paliurus* leaves and tea products [43,44].

To further evaluate the overall differentiation of VOC profiles among harvest months, PCA-X and OPLS-DA were performed (Figure 4). In the PCA-X model, four principal components were extracted, with R^2^X(cum) = 0.743 and Q^2^(cum) = 0.392. The first two plotted components explained 25.1% and 20.5% of the X-variance, respectively. The PCA-X score plot showed a month-dependent distribution tendency, although complete separation among all harvest months was not achieved in this unsupervised model. In particular, Q5, Q7, and Q8 were distributed in relatively distinct regions, whereas Q6 and Q9 were closer to each other, suggesting partial similarity between these two stages in the overall VOC profile.

To further explore the month-associated variation in VOC profiles, an OPLS-DA model was constructed as a supervised exploratory analysis. The score plot showed tendencies of separation among harvest months under supervised conditions, suggesting the presence of month-associated variation within the present dataset. The OPLS-DA model yielded cumulative parameters of R^2^X(cum) = 0.456, R^2^Y(cum) = 0.951, and Q^2^(cum) = 0.874. CV-ANOVA confirmed the statistical significance of the model (F = 9.99521, *p* = 1.68258 × 10^−8^), and the 200-times permutation tests yielded R^2^ intercepts of 0.474–0.499 and Q^2^ intercepts of −0.762 to −0.707, suggesting acceptable robustness under the present exploratory conditions. Given the limited sample size relative to the number of VOC variables, the OPLS-DA results should be interpreted as exploratory multivariate observations rather than as an independent predictive or classification model.

At the chemical-class level, alkenes/terpenes represented the predominant VOC fraction in most harvest months, but their relative contents varied substantially among developmental stages (Figure 5). In Q5, alkenes/terpenes accounted for 71.78% of the total detected volatiles, followed by alkynes (11.67%), alcohols (7.32%), ketones (4.89%), ethers/furans (3.23%), aromatic hydrocarbons (0.76%), and alkanes (0.34%), whereas aldehydes, esters, nitrogen-containing compounds, and other oxygenated compounds were not detected. In Q6, alkenes/terpenes further increased and reached the highest relative abundance among all months (91.49%), while the proportions of ketones (2.59%), alcohols (1.87%), alkynes (1.63%), esters (1.45%), and ethers/furans (0.97%) were relatively low. These results indicate that Q6 was characterized by a strongly alkene/terpene-dominated volatile profile.

In contrast, the VOC profile shifted markedly in Q7. Alkenes/terpenes decreased to 23.39%, whereas alcohols increased to 37.04% and became the most abundant class in this month. Ketones (14.15%), alkynes (10.41%), aldehydes (5.17%), ethers/furans (3.13%), esters (3.03%), alkanes (2.30%), nitrogen-containing compounds (0.76%), and aromatic hydrocarbons (0.61%) were also detected, suggesting a shift from alkene/terpene predominance to a profile characterized by a higher relative peak-area proportion of alcohols and the participation of multiple volatile classes. In Q8, alkenes/terpenes increased again to 41.76% and remained the most abundant class. Meanwhile, alkanes showed a relatively high mean proportion (17.99%), followed by ketones (10.14%), alcohols (9.36%), aldehydes (7.10%), alkynes (5.92%), esters (3.33%), other oxygenated compounds (2.77%), and ethers/furans (1.63%), indicating a more heterogeneous VOC profile with contributions from multiple chemical classes. By Q9, alkenes/terpenes remained the dominant class (64.50%), followed by alcohols (12.03%), alkynes (8.20%), alkanes (5.50%), esters (3.91%), ethers/furans (3.18%), ketones (1.54%), and aldehydes (1.13%). Overall, the VOC profile was characterized by alkene/terpene predominance in Q5–Q6, a higher relative peak-area proportion of alcohols in Q7, a more heterogeneous multi-class profile in Q8, and renewed alkene/terpene predominance in Q9.

At the individual-compound level, tentatively annotated VOCs with relatively stable detection and acceptable repeatability also exhibited clear month-dependent differences (Table 2 and Table S2). In Q5, Dihydroterpineol, 1-Decene, δ-Elemene, dec-2-yne, Dill ether, and cis-Carveol were detected as representative compounds. In Q6, Silphinene and Isocaryophyllene were observed among the representative tentatively annotated terpene-related compounds, together with dec-2-yne, 2-Cyclohexen-1-ol, 3-methyl-6-(1-methylethyl)-acetate, and Dill ether. Q7 showed a higher relative peak-area proportion of several alcohols and related compounds, including 3,3,5-Trimethylcyclohexanol, (-)-Isopulegol, cis-Ocimenol, trans-Sabinene hydrate, 4-Methyl-1-pentanol, and 2-Cyclohexen-1-ol, 3-methyl-6-(1-methylethyl)-acetate. In addition, dec-2-yne was relatively abundant in Q7. In Q8, cis-Ocimenol, Cyclodecane, 2-Cyclohexen-1-ol, 3-methyl-6-(1-methylethyl)-acetate, Dill ether, and cis-Carveol were detected as representative compounds. By Q9, δ-Terpineol, neoiso-3-thujyl acetate, Cyclooctane, 1,4-dimethyl-, cis-Cascarilladiene, (-)-Isopulegol, δ-Elemene, Dill ether, and cis-Carveol were among the representative VOCs. Notably, Dill ether was detected across all five harvest months, whereas many other representative compounds appeared only in specific stages, suggesting qualitative turnover of VOCs during seasonal development. Considering the variability of individual VOC signals, the following discussion focuses mainly on representative compounds with acceptable repeatability rather than on highly variable individual compounds.

### 3.5. Integrated Heatmap and Correlation Analyses of VOC Classes, Bioactive Constituent Indices, and Bioactivity Indices

**Figure 6:**
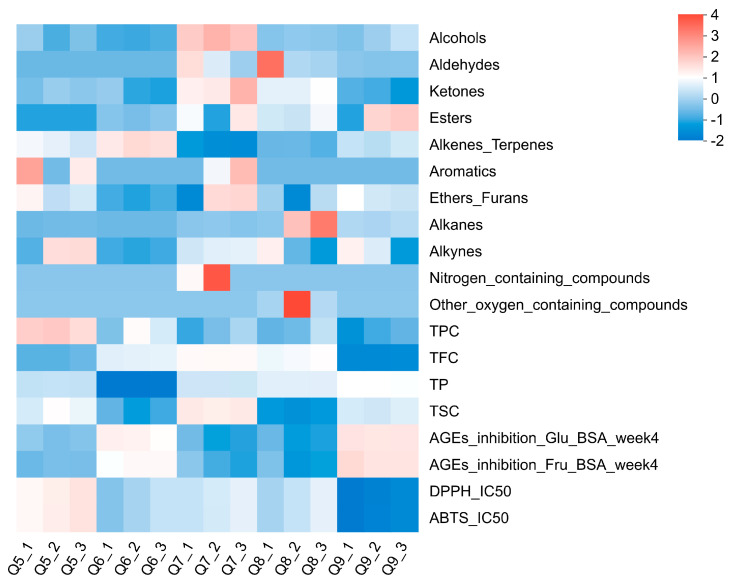
Integrated temporal heatmap of VOC classes, bioactive constituent indices, and bioactivity-related indices in *C. paliurus* leaves across different harvest months. Note: The heatmap was constructed based on row-wise Z-score normalization of VOC classes, bioactive constituent indices, including total phenolic content (TPC), total flavonoid content (TFC), total polysaccharide content (TP), and total saponin content (TSC), and bioactivity-related indices, including the week-4 AGE inhibition rates in the Glu–BSA and Fru–BSA models and the IC_50_ values obtained from the DPPH and ABTS•+ radical scavenging assays. Q5–Q9 represent samples collected from May to September, and 1–3 indicate three biological replicates. For the DPPH and ABTS•+ assays, lower IC_50_ values indicate stronger radical scavenging activity. Red and blue colors indicate relatively higher and lower standardized values, respectively. This heatmap is intended to visualize month-associated temporal co-variation patterns and does not represent formal statistical correlation or causal relationships.

To further explore coordinated temporal patterns among volatile organic compound (VOC) classes, bioactive constituent indices, and bioactivity-related indices of *C. paliurus* leaves, an integrated temporal heatmap was constructed based on row-wise Z-score normalization of VOC classes, total polysaccharides (TP), total saponins (TSC), total polyphenols (TPC), total flavonoids (TFC), and bioactivity-related indices, including antioxidant and anti-glycation activities (Figure 6). For anti-glycation-related indices, the AGE inhibition rates at week 4 in the Glu–BSA and Fru–BSA models were used to represent endpoint inhibitory responses after prolonged incubation, whereas antioxidant-related indices were represented by the IC_50_ values obtained from both the DPPH and ABTS•+ radical scavenging assays. The original IC_50_ values were retained without transformation; therefore, lower IC_50_ values indicate stronger radical scavenging responses. The growth-stage-dependent variations observed in the heatmap suggested temporal divergence in both chemical composition and bioactivity-related indices across the harvest period.

A distinct late-stage bioactivity-related profile was observed in Q9. As shown in Figure 6, Q9 was associated with relatively strong radical scavenging responses, as indicated by the lowest DPPH IC50 values among the tested harvest months. This pattern coincided temporally with relatively higher ester signals and moderate levels of alkenes/terpenes in the volatile profile. In addition, Q9 showed relatively higher AGE inhibition at the tested fixed concentration, especially in the Fru–BSA model, in which the week-4 AGE inhibition rate was higher than that of the other harvest months. These results suggest that the late harvest stage may be associated with enhanced antioxidant capacity and relatively strong anti-glycation potential. However, the specific contribution of individual VOC classes or non-volatile constituents to these bioactivities was not directly resolved in the present study.

In contrast, Q6 showed a different bioactivity-related pattern. The heatmap indicated that Q6 was characterized by a higher relative peak-area proportion of alkenes/terpenes and relatively higher AGE inhibition in the Glu–BSA model at the tested fixed concentration. In the Fru–BSA model, Q6 also showed a relatively strong inhibitory response at the tested fixed concentration, although it was lower than that of Q9. This temporal pattern was consistent with the anti-glycation results described in Section 3.3, indicating that Q6 and Q9 showed relatively stronger AGE inhibitory responses at the tested fixed concentration. Nevertheless, because VOC profiling and anti-glycation assays represent different analytical endpoints, the association between the relative peak-area proportion of alkenes/terpenes and AGE inhibitory responses should be interpreted as temporal co-variation rather than direct evidence of causal contribution.

Interestingly, late-season leaves (September, Q9) showed the lowest IC_50_ values in the DPPH and ABTS•+ assays, despite lower TPC and TFC levels than those observed at some mid-season harvest stages. From a bioclimatic perspective, September represents the transition toward the late growth stage, characterized by decreased temperatures and altered precipitation regimes (as summarized in Table S1). Such shifting environmental factors may act as abiotic cues associated with changes in plant metabolism. One possible explanation is that seasonal environmental changes may be associated with qualitative shifts in phenolic or flavonoid subclasses, which could influence radical scavenging responses. For instance, low temperature and moisture stress may accelerate the deglycosylation of bound flavonoids (e.g., converting flavonoid glycosides into their corresponding aglycones) or the conversion of simple phenolic acids into highly active oligomeric or tannin-like derivatives, thereby increasing the density of sterically unhindered, free phenolic hydroxyl groups per molecule. This interpretation is consistent with previous multi-platform metabolomics observations on *C. paliurus* developmental dynamics [1], which revealed variation in specific antioxidant-related monomers (particularly quercetin derivatives and highly hydroxylated phenolic acids) during the late growth phase, even when the bulk pools of TPC and TFC began to contract. This finding suggests that the tested activities may be influenced not only by the total contents of phenolics or flavonoids but also by qualitative differences in specific phytochemical subclasses, their structural characteristics, and potential interactions among different constituents [40].

In addition, alcohols and ketones were more prominent in Q7, whereas aldehydes, alkanes, and several low-abundance VOC classes showed relatively higher signals or replicate-level variation in Q8; however, these changes did not correspond to the strongest measured bioactivities. The integrated heatmap suggests that month-associated changes in the bioactivity-related indices of *C. paliurus* leaves were accompanied by coordinated but non-identical variation in both bioactive constituent indices and specific VOC classes across harvest stages. These temporally coordinated patterns may provide a preliminary basis for describing functional differentiation among harvest months and for harvest-stage-related utilization, while further targeted validation is still required.

**Figure 7:**
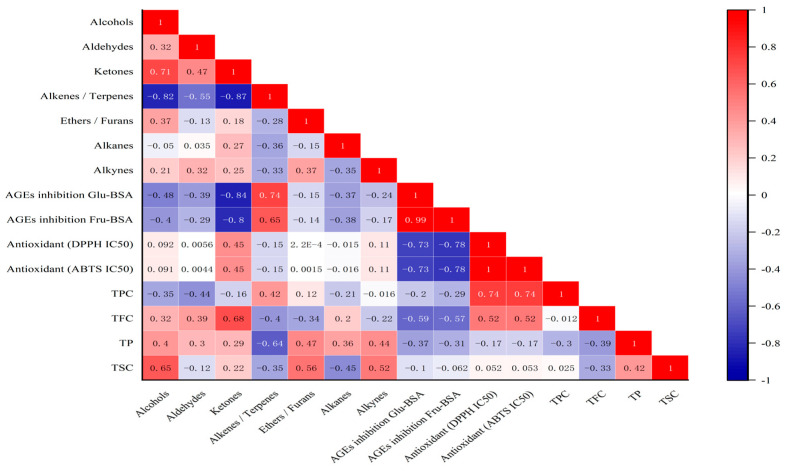
Exploratory Pearson correlation heatmap of selected major volatile compound classes, bioactive constituent indices, and bioactivity indices in *C. paliurus* leaves across different harvest months. Notes: Selected major volatile compound classes with relatively high relative peak-area proportions were included in the analysis, namely alcohols, aldehydes, ketones, alkenes/terpenes, ethers/furans, alkanes, and alkynes. Bioactive constituent indices included total phenolic content (TPC), total flavonoid content (TFC), total polysaccharide content (TP), and total saponin content (TSC). Bioactivity indices included the week-4 inhibition rates of advanced glycation end-product (AGE) formation in the glucose–bovine serum albumin (Glu–BSA) and fructose–bovine serum albumin (Fru–BSA) models, as well as the IC_50_ values obtained from the DPPH and ABTS•+ radical scavenging assays. Values shown in the cells represent Pearson correlation coefficients (r). Red indicates positive correlations, whereas blue indicates negative correlations. The original DPPH and ABTS•+ IC_50_ values were retained without transformation; therefore, lower IC_50_ values indicate stronger radical scavenging activity, and negative correlations with IC_50_ values indicate associations with stronger radical scavenging responses.

Further exploratory Pearson correlation analysis was performed to examine pairwise associations among selected major VOC classes with relatively high relative peak-area proportions, bioactive constituent indices, and bioactivity indices (Figure 7). In this exploratory analysis, the AGE inhibition rates in the Glu–BSA and Fru–BSA models were strongly positively correlated (r = 0.986, *p* < 0.01), while the DPPH and ABTS•+ IC_50_ values also showed a strong positive correlation (r = 1.000, *p* < 0.01). Both AGE inhibition indices were negatively correlated with the original DPPH and ABTS•+ IC_50_ values (r = −0.729 to −0.776, *p* < 0.01). Because lower IC_50_ values indicate stronger radical scavenging activity, these exploratory results suggest that stronger AGE inhibitory responses tended to co-occur with stronger radical scavenging responses under the investigated conditions. In this exploratory analysis, alkenes/terpenes were positively correlated with AGE inhibition in the Glu–BSA and Fru–BSA models (r = 0.736 and 0.651, respectively; both *p* < 0.01), whereas ketones were negatively correlated with these two indices (r = −0.842 and −0.800, respectively; both *p* < 0.01). In addition, TPC was positively correlated with both DPPH IC_50_ and ABTS•+ IC_50_ values (r = 0.743 and 0.744, respectively; both *p* < 0.01), which was consistent with the observation that relatively strong radical scavenging responses at specific harvest stages were not simply associated with higher total phenolic content.

### 3.6. Characterization of Volatile Compounds with Odor Descriptions and Potential Sensory Profile Variation

Beyond the compositional variation revealed by SPME-GC-MS analysis, the month-dependent changes in VOCs may also be associated with corresponding differences in the potential aroma-related profiles of *C. paliurus* leaves. While Figure 5 shows the dynamic remodeling of volatile compound classes across harvest months, Table 3 provides qualitative information on representative volatile compounds with available odor descriptions. Several differential VOCs satisfying VIP > 1.0 and *p* < 0.05 were also included in Table 3 when odor descriptions were available. Therefore, the following interpretation is based on reported odor descriptions and should be regarded as a preliminary indication of potential aroma characteristics rather than direct sensory evidence.

In the early harvest stages (Q5–Q6), the potential aroma-related profile was mainly associated with alkene/terpene-related woody, herbal, spicy, pine-like, citrus, floral, green, and slightly waxy notes. In Q5, alkenes/terpenes were the predominant volatile class, accompanied by alkynes, alcohols, ketones, and ethers/furans at lower proportions. Representative odor-described compounds in Q5, including Dihydroterpineol, 1-Decene, δ-Elemene, Dill ether, cis-Carveol, α-Cubebene, β-Damascone, α-Fenchene, and (-)-trans-Pinocarveol, were associated with pine, lime, citrus, floral, herbal, waxy, woody, spicy, camphoraceous, warm, and balsamic odor notes. In Q6, alkenes/terpenes became overwhelmingly dominant, indicating a more pronounced terpene-dominated volatile profile. Representative compounds such as Silphinene, Isocaryophyllene, 2-Cyclohexen-1-ol, 3-methyl-6-(1-methylethyl)-, acetate, Dill ether, and α-Cubebene were mainly associated with woody, spicy, herbal, green, fresh, and waxy notes. These results suggest that Q5–Q6 may represent an alkene/terpene-associated aroma stage, with Q6 showing stronger terpene-dominated characteristics.

During the middle harvest stage Q7, the potential aroma-related profile became more diversified, corresponding to a higher relative peak-area proportion of alcohols and the increased contribution of ketones, alkynes, aldehydes, esters, and ethers/furans. Representative compounds detected in Q7, including 3,3,5-Trimethylcyclohexanol, (-)-Isopulegol, trans-Sabinene hydrate, 4-Methyl-1-pentanol, 2-Cyclohexen-1-ol, 3-methyl-6-(1-methylethyl)-, acetate, (+)-Aromadendrene, Dill ether, cis-Carveol, trans-Isopiperitenol, and cis-Ocimenol, were associated with musty, cooling, minty, medicinal, green, herbal, citrus, spicy, fresh, woody, nutty, floral, sweet, and mace-like odor notes. Compared with Q5–Q6, Q7 therefore showed a shift toward a fresher, greener, mint-like, and more layered potential aroma profile.

In Q8, the volatile profile was characterized by a more heterogeneous multi-class distribution. Although alkenes/terpenes remained the largest VOC class, alkanes, ketones, alcohols, aldehydes, alkynes, esters, and other oxygen-containing compounds also contributed to the overall volatile profile. The odor-described compounds detected in this month, including 2-Cyclohexen-1-ol, 3-methyl-6-(1-methylethyl)-, acetate, Dill ether, cis-Carveol, Cyclodecane, 2-Cyclohexen-1-ol, 2-methyl-5-(1-methylethenyl)-, (1R,5S)-rel-, and cis-Ocimenol, were associated with green, herbal, fresh, spicy, citrus, minty, sweet, musk-like, dry, amber, and woody notes. These observations indicate that Q8 may correspond to a more heterogeneous aroma-related stage, reflecting its broader multi-class volatile composition.

In the late harvest stage Q9, alkenes/terpenes again became the dominant volatile class, accompanied by alcohols, alkynes, alkanes, esters, ethers/furans, ketones, and aldehydes at lower proportions. Compared with the middle harvest stages, Q9 showed a mixed volatile profile with renewed terpene-related features and relatively noticeable ester signals. The odor-described compounds detected in Q9, including δ-Elemene, (-)-Isopulegol, Dill ether, cis-Carveol, δ-Terpineol, 10-Undecenal, α-Cubebene, and trans-Isopiperitenol, were associated with sweet, herbal, woody, minty, cooling, medicinal, green, spicy, pine-like, floral, lime-like, mildly waxy, rosy-citrusy, fruity, and fresh notes. These results suggest that Q9 exhibited a mixed potential aroma profile, with renewed terpene-related characteristics together with sweet, herbal, floral, citrus-like, and mildly waxy odor descriptors.

Overall, the odor-description-based analysis suggests a month-dependent transition in the potential aroma-related properties of *C. paliurus* leaves. Q5–Q6 were mainly associated with alkene/terpene-related woody, herbal, spicy, pine-like, citrus, floral, and fresh-green notes; Q7 showed a fresher, greener, mint-like, and more diversified profile; Q8 displayed a more heterogeneous multi-class aroma-related pattern; and Q9 showed a mixed profile with renewed terpene-related characteristics and relatively noticeable ester-associated features. These findings provide a preliminary reference for harvest-stage-oriented utilization of mature *C. paliurus* leaves. Nevertheless, because odor activity values, gas chromatography–olfactometry, and sensory validation were not determined in the present study, the specific contribution of individual compounds to aroma perception requires further confirmation through dedicated aroma-active and sensory evaluation approaches.

## 4. Discussion

**Figure 8:**
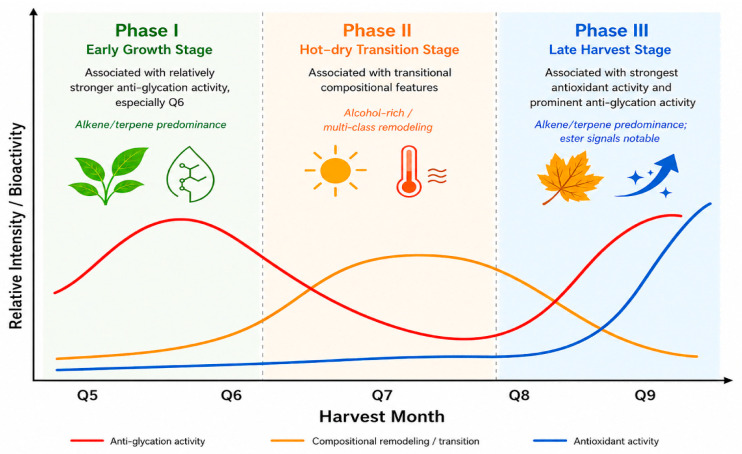
Proposed schematic summary of the three-phase seasonal transition pattern observed in mature *C. paliurus* leaves from Q5 to Q9 in this single-site, single-year study. Note: Phase I (Q5–Q6) was associated with alkene/terpene predominance and relatively stronger AGE inhibitory responses among the tested leaf extracts at the single fixed concentration, especially in Q6, under the investigated conditions. Phase II (Q7–Q8) represented a hot–dry transitional stage with compositional remodeling, including a higher relative peak-area proportion of alcohols in Q7 and a heterogeneous multi-class VOC profile in Q8. Phase III (Q9) was associated with relatively strong radical scavenging responses in both the DPPH and ABTS•+ assays and relatively strong AGE inhibitory responses among the tested leaf extracts at the single fixed concentration, especially in the Fru–BSA model, together with renewed alkene/terpene predominance and relatively noticeable ester signals. The curves are schematic and represent relative trends rather than fitted quantitative values.

Based on the integrated analysis of VOC classes, bioactive constituent indices, bioactivity indices, and monthly meteorological conditions (Figure 8), the seasonal variation observed in this single-site, single-year dataset may be tentatively described as a three-phase transition pattern rather than a simple linear maturation process. The integrated temporal heatmap (Figure 6) showed that different harvest months were associated with distinct combinations of VOC profiles, bioactive constituent indices, radical scavenging responses, and AGE inhibitory responses. Complementing these temporal patterns, exploratory Pearson correlation analysis (Figure 7) indicated that stronger AGE inhibitory responses tended to co-occur with stronger radical scavenging responses, as reflected by the negative correlations between AGE inhibition rates and the original DPPH and ABTS•+ IC_50_ values. Among the selected major VOC classes, alkenes/terpenes showed positive pairwise associations with AGE inhibition, whereas ketones showed negative pairwise associations with these anti-glycation indices in the exploratory analysis. These correlations represent exploratory pairwise associations and should be interpreted cautiously, without implying direct causal relationships. Meanwhile, the monthly meteorological data (Supplementary Table S1) indicated clear month-to-month variation in rainfall, rainy days, and temperature during the sampling period. In particular, Q6 was characterized by the highest rainfall and the greatest number of rainy days, whereas Q7 and Q8 corresponded to the warmest and relatively driest period. Q9 showed a decline in temperature compared with Q7–Q8, together with increased rainfall and rainy days, suggesting a shift toward a later seasonal stage. These meteorological changes were temporally associated with the observed shifts in volatile composition and bioactivity-related indices during leaf development, although direct causal relationships were not established in the present study. The proposed three-phase framework should therefore be regarded as a descriptive interpretation of this single-site, single-year dataset rather than a generalized developmental model for *C. paliurus*.

Phase I (Q5–Q6) may represent an early-stage pattern in the present dataset, characterized by alkene/terpene predominance and relatively stronger AGE inhibitory responses at the tested fixed concentration, especially in Q6. At the VOC class level, Q5 and Q6 were both dominated by alkenes/terpenes, with Q6 showing the highest relative abundance of this class among all harvest months. This interpretation was consistent with the exploratory correlation analysis, in which alkenes/terpenes showed positive pairwise associations with AGE inhibition in both the Glu–BSA and Fru–BSA models, whereas ketones showed negative pairwise associations with these anti-glycation indices. This pattern was also reflected in the odor-description-based analysis, in which Q5–Q6 were mainly associated with alkene/terpene-related woody, herbal, spicy, pine-like, citrus, floral, and fresh-green notes. From a functional perspective, the anti-glycation results showed that Q6 and Q9 exhibited relatively stronger AGE inhibitory responses among the tested leaf extracts at the single fixed concentration, although their model-dependent patterns were not identical. In the Glu–BSA model, Q6 and Q9 showed relatively high inhibition rates from week 2 onward; however, the single fixed-concentration design does not allow direct potency comparison or equivalence assessment with AG. In the Fru–BSA model, Q9 showed a relatively higher AGE inhibitory response among the leaf extracts at the tested fixed concentration, followed by Q6, whereas AG exhibited a significantly higher inhibition rate than all leaf extracts at week 4. Therefore, under the single fixed-concentration assay conditions, Q6 may be interpreted as an early-stage sample showing relatively strong AGE inhibitory responses among the tested leaf extracts, without implying a true potency ranking, equivalence to AG, or a generally optimal harvest stage. Previous studies have indicated that plant polyphenols and flavonoids may inhibit glycation through multiple pathways, including antioxidant-related effects and interactions with reactive carbonyl intermediates [45,46]. However, the present data support only a harvest month-associated pattern and do not provide direct mechanistic evidence linking specific volatile changes to AGE inhibitory responses. From an environmental perspective, the relatively mild and humid conditions observed in Q6 may have been associated with active vegetative growth and metabolite accumulation during this period.

Phase II (Q7–Q8) may be regarded as a hot–dry transitional stage characterized by compositional remodeling. According to the meteorological data, July and August showed the highest maximum temperatures and the fewest rainy days, indicating a warmer and comparatively drier environment than the other sampling months. In parallel, the VOC profile shifted markedly from alkene/terpene predominance to a more diversified composition. Q7 was characterized by a higher relative peak-area proportion of alcohols, accompanied by increased contributions of ketones, alkynes, aldehydes, esters, and ethers/furans. Q8 showed a more heterogeneous multi-class VOC profile, with alkenes/terpenes remaining the most abundant class but with notable contributions from alkanes, ketones, alcohols, aldehydes, alkynes, esters, and other oxygen-containing compounds. In terms of bioactive constituent indices, TFC was relatively higher during Q7–Q8. However, these compositional changes did not correspond to the strongest measured bioactivity-related responses. Radical scavenging responses in Q7–Q8 were weaker than those in Q9, and AGE inhibitory responses at the tested fixed concentration were generally lower than those observed for Q6 and Q9. Taken together, these findings suggest that Q7–Q8 may represent an intermediate seasonal stage characterized by phytochemical and volatile remodeling rather than by the strongest responses in either tested bioactivity-related assay.

Phase III (Q9) may represent the late harvest stage in the present dataset and was associated with relatively strong radical scavenging responses in both the DPPH and ABTS•+ assays, together with relatively strong AGE inhibitory responses among the tested leaf extracts at the single fixed concentration, especially in the Fru–BSA model. Q9 exhibited the lowest IC_50_ value for DPPH radical scavenging activity, indicating a relatively strong radical scavenging response among the five harvest months under the investigated conditions. Notably, these relatively strong radical scavenging responses did not coincide with the highest total flavonoid content or total phenolic content, which were mainly observed in Q7–Q8 and Q5, respectively. Consistent with this observation, the exploratory correlation analysis showed positive associations between TPC and the original DPPH and ABTS•+ IC_50_ values, suggesting that stronger radical scavenging responses were not simply associated with higher total phenolic content. This suggests that the radical scavenging responses of *C. paliurus* leaf extracts may not be associated solely with the total contents of phenolics or flavonoids, but may also reflect qualitative differences in specific antioxidant-related constituents, their structural characteristics, and possible interactions among different phytochemical groups. In terms of VOC composition, Q9 showed renewed alkene/terpene predominance, together with alcohols, alkynes, alkanes, esters, ethers/furans, ketones, and aldehydes at lower proportions. Compared with Q7–Q8, Q9 also exhibited relatively noticeable ester-related signals, which was consistent with the mixed potential aroma-related profile described in Section 3.6. Therefore, under the investigated conditions, Q9 was characterized by relatively strong radical scavenging responses in both assays, relatively strong AGE inhibitory responses among the tested leaf extracts at the single fixed concentration, and a mixed volatile profile with renewed terpene-related and ester-associated features.

Collectively, the present results suggest a tentative three-phase seasonal transition pattern in mature *C. paliurus* leaves within this single-site, single-year dataset. Phase I (Q5–Q6) was characterized by alkene/terpene predominance and relatively stronger AGE inhibitory responses at the tested fixed concentration, especially in Q6. Phase II (Q7–Q8) represented a hot–dry transitional stage with marked compositional remodeling, including a higher relative peak-area proportion of alcohols in Q7 and a more heterogeneous multi-class VOC profile in Q8. Phase III (Q9) was associated with relatively strong radical scavenging responses in both the DPPH and ABTS•+ assays and relatively strong AGE inhibitory responses among the tested leaf extracts at the single fixed concentration, especially in the Fru–BSA model, together with renewed alkene/terpene predominance and relatively noticeable ester signals. This framework should be regarded as an interpretive pattern derived from this single-site, single-year dataset, rather than as direct evidence of specific biochemical or physiological mechanisms or as a generalized developmental model for *C. paliurus*.

It should also be noted that the present study employed an aqueous extraction system to simulate the practical consumption of *C. paliurus* leaves as an infusion. Therefore, the bioactivity results mainly reflect the functional characteristics of water extracts under the investigated conditions, whereas volatile profiling was evaluated separately at the dried leaf powder level. Accordingly, the exploratory correlations observed between selected VOC classes and bioactivity-related indices should be interpreted as coordinated harvest month-associated associations rather than direct evidence that volatile constituents contributed to the measured radical scavenging or AGE inhibitory responses.

Moreover, the VOC annotations in this study were based on NIST library matching combined with RI comparison and should be considered tentative identifications. The relative contents of VOCs were estimated by peak-area normalization and therefore represent semi-quantitative profiles. In addition, odor descriptions were obtained from reported databases or literature sources, while odor activity values, gas chromatography-olfactometry, and sensory validation were not determined. Thus, the aroma-related interpretation should be considered a qualitative indication of potential sensory attributes rather than direct evidence of aroma contribution. Finally, all samples were collected from a single site within one growing season and were subjected to the same drying and powdering procedures. Accordingly, the present results should be interpreted as preliminary, site- and year-specific observations under the investigated field and processing conditions. Future studies incorporating reference standards, targeted compound quantification, multi-site and multi-year sampling, aroma-active validation, and mechanistic experiments will be needed to further evaluate the robustness and generalizability of the present observations.

## 5. Conclusions

This study showed that *C. paliurus* leaves harvested in different months exhibited month-associated variation in VOC composition and bioactivity-related properties under the investigated conditions. Antioxidant evaluation using two complementary radical scavenging assays indicated that Q9 exhibited relatively strong radical scavenging responses in both the DPPH and ABTS•+ assays, while Q6 also showed comparatively strong radical scavenging responses, particularly in the DPPH assay. In the anti-glycation evaluation, Q6 and Q9 displayed relatively stronger AGE inhibitory responses among the tested leaf extracts at the single fixed concentration under the investigated conditions. The VOC profiles further showed marked month-associated differences, including alkene/terpene predominance in Q5–Q6, a higher relative peak-area proportion of alcohols in Q7, a heterogeneous multi-class profile in Q8, and renewed alkene/terpene predominance in Q9 accompanied by relatively noticeable ester signals. Exploratory Pearson correlation analysis further revealed pairwise associations among selected major VOC classes, bioactive constituent indices, and bioactivity indices. In particular, within the present dataset, stronger AGE inhibitory responses tended to coincide with lower DPPH and ABTS•+ IC_50_ values, suggesting coordinated but exploratory patterns between AGE inhibition and radical scavenging responses under the investigated conditions. These associations should be interpreted cautiously, particularly given the exploratory nature of the correlation analysis and the single-site, single-year design, and do not demonstrate direct causal relationships. In addition, because the anti-glycation assay was conducted at a single fixed concentration, the observed inhibitory responses should not be interpreted as a complete potency ranking or as equivalence to AG.

Overall, these findings may be tentatively interpreted as a descriptive three-phase month-associated pattern in mature *C. paliurus* leaves within this single-site, single-year dataset, comprising an early-stage pattern characterized by alkene/terpene predominance and relatively stronger AGE inhibitory responses among the tested leaf extracts at the single fixed concentration, especially in Q6; an intermediate hot–dry transitional stage characterized by compositional remodeling during Q7–Q8; and a late-harvest stage in which Q9 exhibited relatively strong radical scavenging responses in both the DPPH and ABTS•+ assays and relatively strong AGE inhibitory responses among the tested leaf extracts at the single fixed concentration, especially in the Fru–BSA model. Because sampling was conducted at a single site within one growing season, the present results should be interpreted as preliminary, site- and year-specific observations under the investigated conditions. Nevertheless, these findings may provide a preliminary reference for harvest-stage-oriented utilization of mature *C. paliurus* leaves and for the design of broader multi-site, multi-year validation studies.

## Figures and Tables

**Table 1:** IC_50_ values for DPPH and ABTS•+ radical scavenging activity of *C. paliurus* leaves harvested in five different months.

Sample	DPPH IC_50_ (mg/mL)	ABTS•+ IC_50_ (mg/mL)
Q5	0.297 ± 0.01 ^a^	0.327 ± 0.01 ^a^
Q6	0.222 ± 0.02 ^c^	0.244 ± 0.02 ^c^
Q7	0.252 ± 0.01 ^b^	0.277 ± 0.01 ^b^
Q8	0.242 ± 0.02 ^bc^	0.266 ± 0.02 ^bc^
Q9	0.119 ± 0.01 ^d^	0.131 ± 0.01 ^d^
Vitamin C (Positive control)	0.025 ± 0.002	0.048 ± 0.003

Note: Data are expressed as mean ± SD (n = 3). Different superscript lowercase letters within the same column indicate significant differences among aqueous extracts from different harvest months (*p* < 0.05, Duncan’s multiple range test). Vitamin C was included as the positive control and is presented for reference only; it was not included in the statistical comparison among harvest months. IC_50_ values were calculated independently for the DPPH and ABTS•+ radical scavenging assays by nonlinear regression analysis.

**Table 2:** Major tentatively annotated volatile compounds in *C. paliurus* leaves harvested at different developmental stages (Q5–Q9).

Compound Name	CAS No.	Q5	Q6	Q7	Q8	Q9
Dihydroterpineol	21129-27-1	3.04 ± 0.15	—	—	—	—
1-Decene	872-05-9	2.50 ± 0.62	—	—	—	—
δ-Elemene	20307-84-0	2.10 ± 0.29	—	—	—	1.93 ± 0.17
Silphinene	74284-57-4	—	6.27 ± 1.71	—	—	—
Isocaryophyllene	118-65-0	—	2.06 ± 0.36	—	—	—
dec-2-yne	2384-70-5	1.79 ± 0.17	1.49 ± 0.16	9.28 ± 0.67	—	—
3,3,5-Trimethylcyclohexanol	116-02-9	—	—	9.12 ± 0.25	—	—
(-)-Isopulegol	89-79-2	—	—	5.67 ± 0.37	—	1.99 ± 0.10
cis-Ocimenol	7643-59-6	—	—	5.62 ± 0.67	3.64 ± 1.06	—
trans-Sabinene hydrate	17699-16-0	—	—	4.54 ± 1.14	—	—
4-Methyl-1-pentanol	626-89-1	—	—	4.08 ± 1.05	—	—
2-Cyclohexen-1-ol, 3-methyl-6-(1-methylethyl)-acetate	1204-30-4	—	1.35 ± 0.16	4.01 ± 0.11	2.31 ± 0.14	—
(+)-Aromadendrene	489-39-4	—	—	3.56 ± 0.51	—	—
Dill ether	74410-10-9	2.37 ± 0.29	0.89 ± 0.11	2.60 ± 0.02	1.85 ± 0.06	1.22 ± 0.08
cis-Carveol	1197-06-4	0.28 ± 0.02	—	2.19 ± 0.04	1.67 ± 0.17	1.00 ± 0.13
Cyclodecane	293-96-9	—	—	—	3.30 ± 0.37	—
δ-Terpineol	7299-42-5	—	—	—	—	6.90 ± 1.50
Neoiso-3-thujyl acetate	62181-91-3	—	—	—	—	5.33 ± 0.38
Cyclooctane, 1,4-dimethyl-	13151-99-0	—	—	—	—	4.39 ± 0.21
cis-Cascarilladiene	59742-39-1	—	—	—	—	2.77 ± 0.31

Note: Data are presented as mean ± SD of relative peak-area proportions (%) based on peak-area normalization (n = 3). A dash (—) indicates that the compound was not detected or did not satisfy the inclusion criteria in the corresponding harvest month. The complete list of volatile compounds is provided in Supplementary Table S2.

**Table 3:** Representative potential aroma-related volatile compounds with reported odor descriptions in *C. paliurus* leaves harvested at different developmental stages (Q5–Q9).

Harvest Month	CAS No.	Compound Name	Molecular Formula	Reported Odor Description
Q5	21129-27-1	Dihydroterpineol	C_10_H_20_O	Pine, lime, citrus, floral, terpenic
	872-05-9	1-Decene	C_10_H_20_	Waxy, oily, citrus, fresh
	20307-84-0	δ-Elemene	C_15_H_24_	Sweet, herbal, woody
	74410-10-9	Dill ether	C_10_H_16_O	Dill, herbal
	1197-06-4	cis-Carveol	C_10_H_16_O	Spicy
	17699-14-8	α-Cubebene	C_15_H_24_	Herbal, waxy
	35044-68-9	β-Damascone	C_13_H_20_O	Fruity, floral, fresh, green, woody, rose-like, plum, honey, tobacco, blackcurrant
	471-84-1	α-Fenchene	C_10_H_16_	Camphoraceous
	547-61-5	(-)-trans-Pinocarveol	C_10_H_16_O	Warm, woody, balsamic, fennel
Q6	74284-57-4	Silphinene	C_15_H_24_	Woody-to-floral
	118-65-0	Isocaryophyllene	C_15_H_24_	Woody, spicy
	1204-30-4	2-Cyclohexen-1-ol, 3-methyl-6-(1-methylethyl)-, acetate	C_12_H_20_O_2_	Green, herbal, fresh
	74410-10-9	Dill ether	C_10_H_16_O	Dill, herbal
	17699-14-8	α-Cubebene	C_15_H_24_	Herbal, waxy
Q7	116-02-9	3,3,5-Trimethylcyclohexanol	C_9_H_18_O	Musty, cooling, minty, spicy
	89-79-2	(-)-Isopulegol	C_10_H_18_O	Minty, cooling, medicinal, woody, green, herbal
	17699-16-0	trans-Sabinene hydrate	C_10_H_18_O	Minty, eucalyptus, green, terpenic, spicy
	626-89-1	4-Methyl-1-pentanol	C_6_H_14_O	Nutty
	1204-30-4	2-Cyclohexen-1-ol, 3-methyl-6-(1-methylethyl)-, acetate	C_12_H_20_O_2_	Green, herbal, fresh
	489-39-4	(+)-Aromadendrene	C_15_H_24_	Woody, herbal, spicy, fresh, green
	74410-10-9	Dill ether	C_10_H_16_O	Dill, herbal
	1197-06-4	cis-Carveol	C_10_H_16_O	Spicy
	74410-00-7	trans-Isopiperitenol	C_10_H_16_O	Herbal, mint, green, fruity, floral, spicy, sweet, fresh, woody
	7643-59-6	cis-Ocimenol	C_10_H_18_O	Fresh citrus, lemon, lime, cologne, sweet, mace-like
Q8	1204-30-4	2-Cyclohexen-1-ol, 3-methyl-6-(1-methylethyl)-, acetate	C_12_H_20_O_2_	Green, herbal, fresh
	74410-10-9	Dill ether	C_10_H_16_O	Dill, herbal
	1197-06-4	cis-Carveol	C_10_H_16_O	Spicy
	293-96-9	Cyclodecane	C_10_H_20_	Sweet, musk, dry, amber, woody
	1197-07-5	2-Cyclohexen-1-ol, 2-methyl-5-(1-methylethenyl)-, (1R,5S)-rel-	C_10_H_16_O	Spicy; carveol-like, minty/spearmint/caraway
	7643-59-6	cis-Ocimenol	C_10_H_18_O	Fresh citrus, lemon, lime, cologne, sweet, mace-like
Q9	20307-84-0	δ-Elemene	C_15_H_24_	Sweet, herbal, woody
	89-79-2	(-)-Isopulegol	C_10_H_18_O	Minty, cooling, medicinal, woody, green, herbal
	74410-10-9	Dill ether	C_10_H_16_O	Dill, herbal
	1197-06-4	cis-Carveol	C_10_H_16_O	Spicy
	7299-42-5	δ-Terpineol	C_10_H_18_O	Fresh, clean, woody, pine, floral, lime
	112-45-8	10-Undecenal	C_11_H_20_O	Powerful, mildly waxy, rosy-citrusy
	17699-14-8	α-Cubebene	C_15_H_24_	Herbal, waxy
	74410-00-7	trans-Isopiperitenol	C_10_H_16_O	Herbal, mint, green, fruity, floral, spicy, sweet, fresh, woody

Notes: This table lists representative volatile compounds with available odor descriptions detected in *C. paliurus* leaves collected in different harvest months. Several differential VOCs satisfying VIP > 1.0 and *p* < 0.05 were also included if odor descriptions were available. “Reported odor description” refers to odor characteristics documented in relevant databases or literature sources and should be interpreted as a qualitative indication of potential aroma-related attributes rather than direct evidence of confirmed aroma-active compounds.

## Data Availability

The original contributions presented in the study are included in the article/Supplementary Materials. Further inquiries can be directed to the corresponding author.

## Supplementary Materials

The following supporting information can be downloaded at https://www.mdpi.com/article/10.3390/foods15122183/s1: Table S1: Monthly meteorological conditions and extraction yields at Shimen (Hunan, China) during the sampling period (May–September 2024); Table S2: Tentatively annotated volatile organic compounds in *C. paliurus* leaves harvested at different developmental stages (Q5–Q9), with retention index verification, together with their chemical classes, CAS numbers, relative peak-area proportions (%), retention times (RTs), retention indices (RIs), and relative standard deviations (RSDs); Table S3: Supplementary data set used to generate Figure 2A: concentration-dependent DPPH radical scavenging activities of aqueous extracts from *C. paliurus* leaves harvested in different months; Table S4: Individual fluorescence intensity data for advanced glycation end-product formation in the Glu–BSA and Fru–BSA models used for the anti-glycation evaluation of *C. paliurus* leaf extracts; Table S5: Summary of volatile organic compounds (VOCs) detected in *C. paliurus* leaves that appeared in at least two harvest months, including retention indices (RI) and peak areas; Figure S1: Permutation test plots for the OPLS-DA model based on volatile organic compound profiles of *C. paliurus* leaves harvested in different months; Figure S2: Total ion chromatograms (TICs) obtained by SPME–GC–MS analysis of *C. paliurus* leaf powder samples collected from May to September.

## Abbreviations

Total Phenolic Content (TPC); Total Flavonoid Content (TFC); Total Polysaccharide Content (TP); Total Saponin Content (TSC); Solid-Phase Microextraction Coupled with Gas Chromatography-Mass Spectrometry (SPME–GC–MS); Volatile Organic Compounds (VOCs); 2,2-Diphenyl-1-picrylhydrazyl (DPPH); Bovine Serum Albumin (BSA); Glucose–Bovine Serum Albumin (Glu–BSA); Fructose–Bovine Serum Albumin (Fru–BSA); Advanced Glycation End-Products (AGEs); Aminoguanidine Hydrochloride (AG); Half-Maximal Inhibitory Concentration (IC50); Phosphate-Buffered Saline (PBS); Gas Chromatography–Mass Spectrometry (GC–MS); Solid-Phase Microextraction (SPME); Electron Ionization (EI); Retention Time (RT); Retention Index (RI); Relative Standard Deviation (RSD); Standard Deviation (SD); Principal Component Analysis (PCA); Orthogonal Partial Least Squares Discriminant Analysis (OPLS–DA); National Institute of Standards and Technology (NIST); Analysis of Variance (ANOVA); Cross-Validation Analysis of Variance (CV-ANOVA); Aminoguanidine (AG); Vitamin C (VC); Streptozotocin (STZ); Superoxide Dismutase (SOD); Dry Weight (DW); Traditional Chinese Medicine (TCM).
