# Supplementary material for: Growth Month-Associated Variation in Volatile Profiles, Anti-Glycation Capacity, and Antioxidant Activity of Cyclocarya paliurus Leaves: A Pilot Study
Source: Foods. 2026 Jun 17;15(12):2183. doi: 10.3390/foods15122183 (PMC13298220; doi:10.3390/foods15122183)
Supplement: Supplementary file 1 [file foods-15-02183-s001.zip › foods-4343343-supplementary.pdf]

Supplementary Figure S

(A) Q5

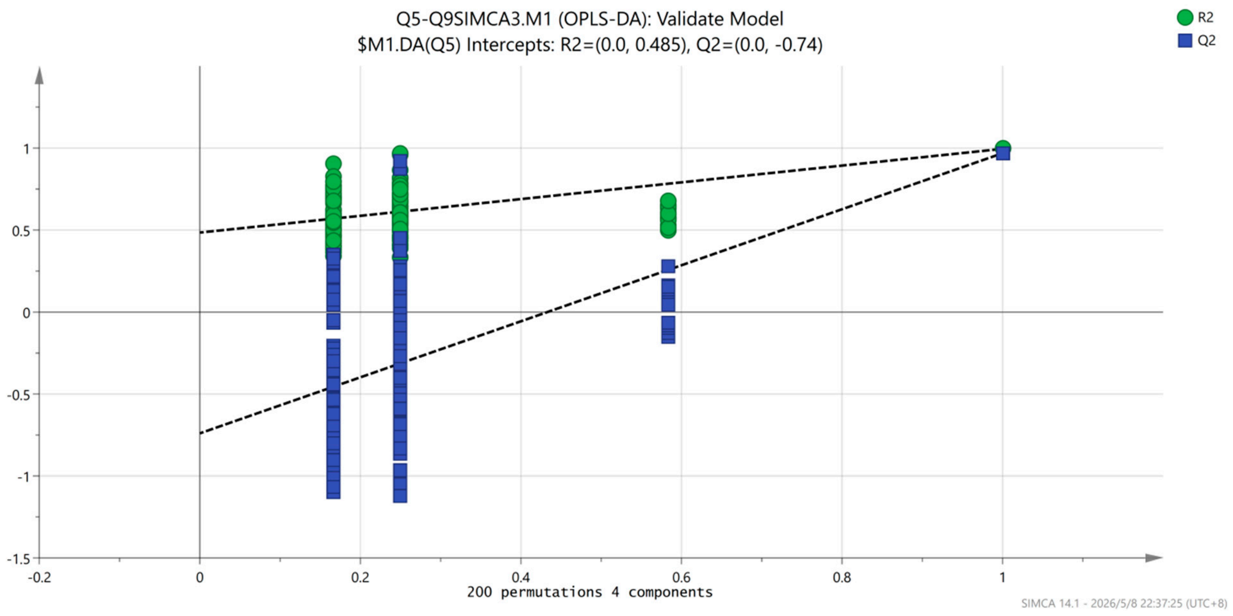

(B) Q6

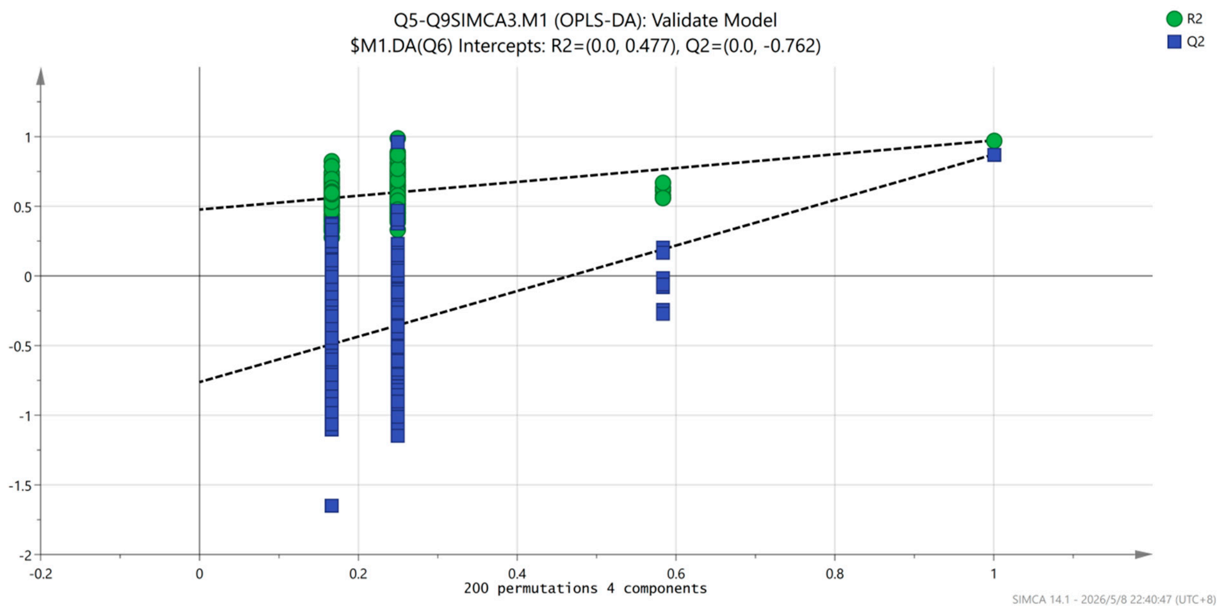

(C) Q7

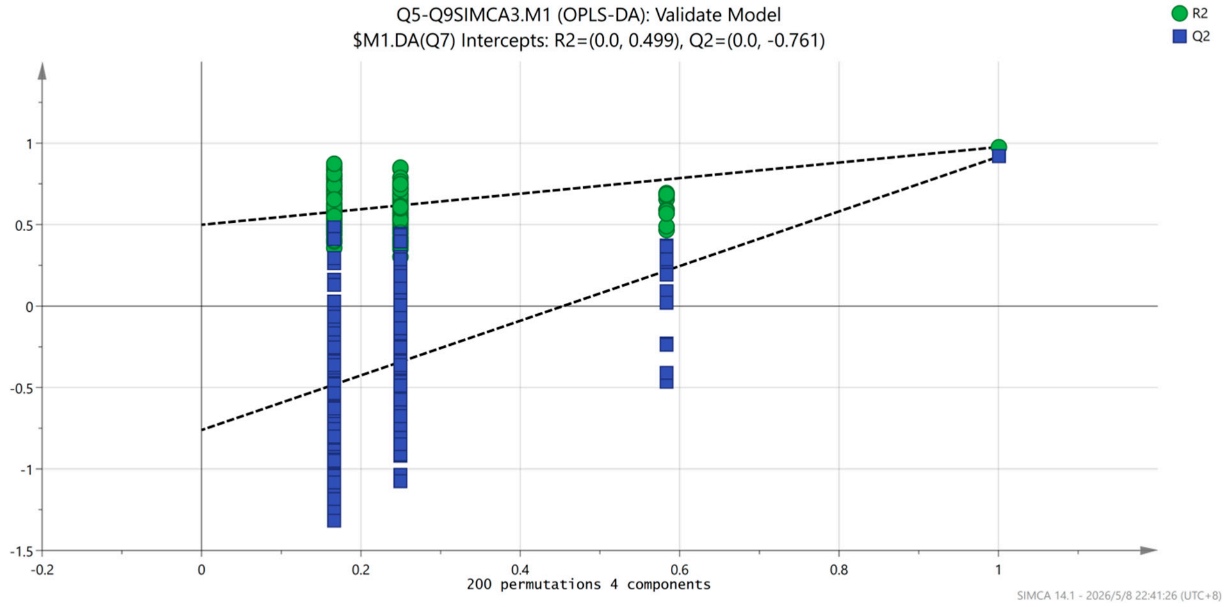

Q5-Q9SIMCA3.M1 (OPLS-DA): Validate Model
$M1.DA(Q7) Intercepts: R2=(0.0, 0.499), Q2=(0.0, -0.761)
R2
Q2
1
0.5
0
-0.5
-1
-1.5
-0.2
0
0.2
0.4
0.6
0.8
1
200 permutations 4 components
SIMCA 14.1 - 2026/5/8 22:41:26 (UTC+8)

(D) Q8

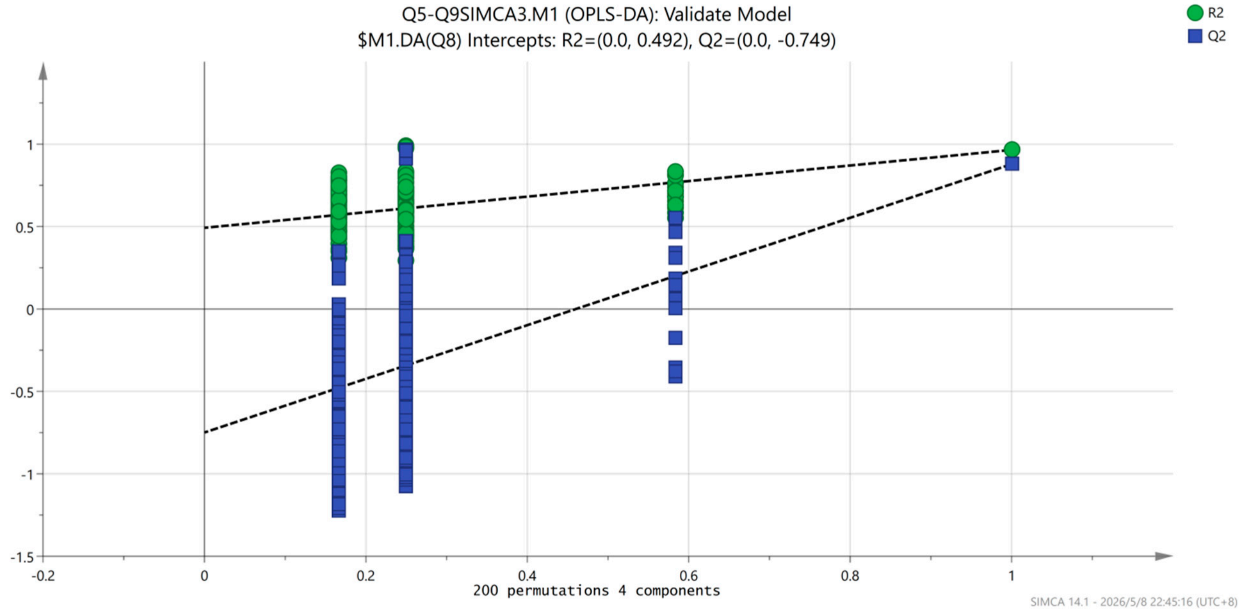

Q5-Q9SIMCA3.M1 (OPLS-DA): Validate Model
$M1.DA(Q8) Intercepts: R2=(0.0, 0.492), Q2=(0.0, -0.749)
R2
Q2
1
0.5
0
-0.5
-1
-1.5
-0.2
0
0.2
0.4
0.6
0.8
1
200 permutations 4 components
SIMCA 14.1 - 2026/5/8 22:45:16 (UTC+8)

(E) Q9

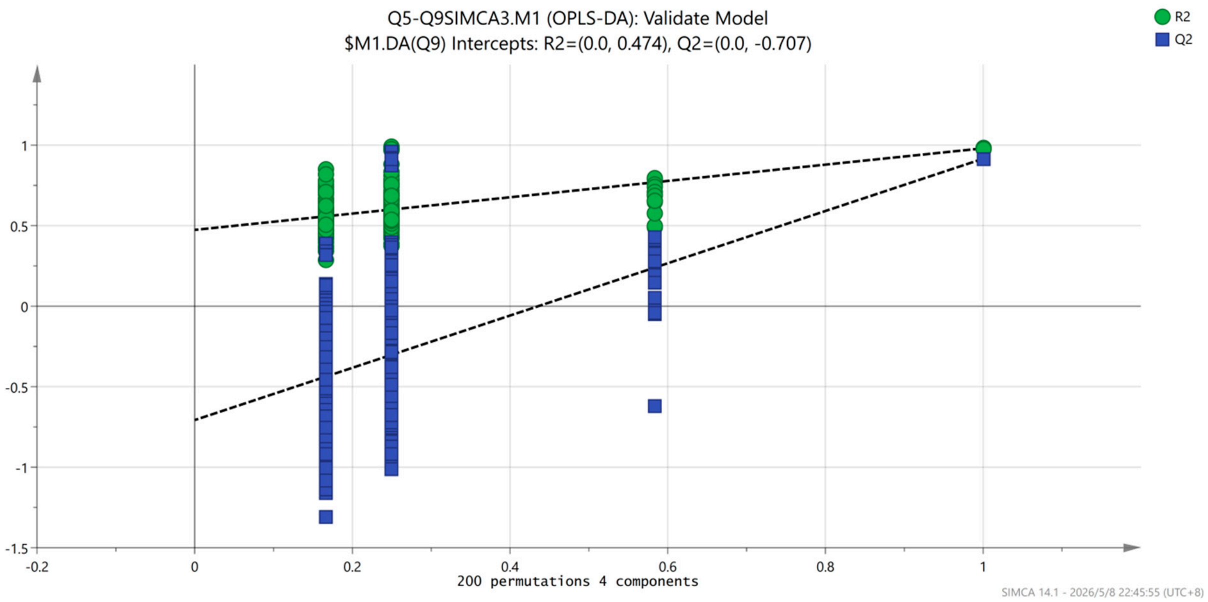

Figure S1. Permutation test plots for the OPLS-DA model based on volatile organic compound profiles of *C. paliurus* leaves harvested in different months. Note: The OPLS-DA model was validated using 200 permutation tests for each harvest-month class: (A) Q5, (B) Q6, (C) Q7, (D) Q8, and (E) Q9. Green circles represent R^2 values, and blue squares represent Q^2 values. The R^2 intercepts ranged from 0.474 to 0.499, whereas the Q^2 intercepts ranged from −0.762 to −0.707. Q5–Q9 denote *C. paliurus* leaf samples collected from May to September, respectively.

(A) Q5

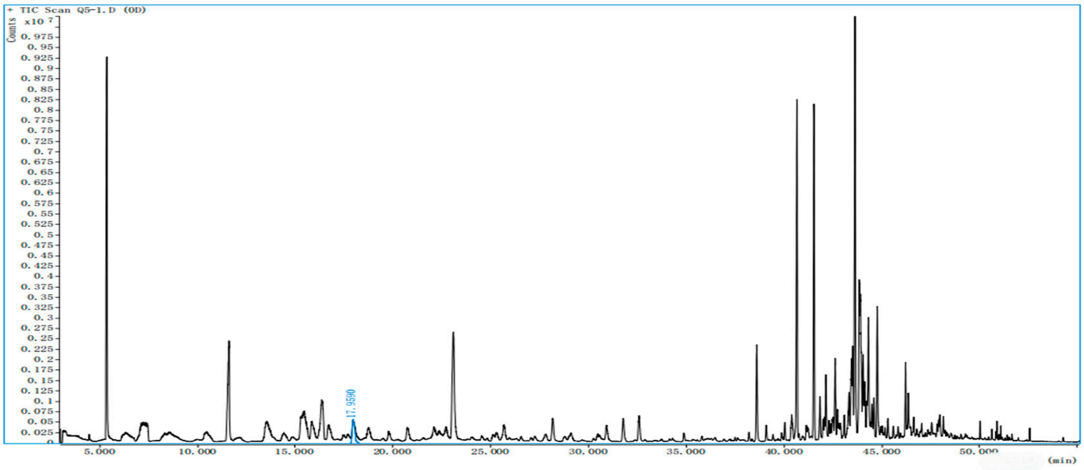
+ TIC Scan Q5-1.D (OD)
Counts
x10 7
17.9590
(min)

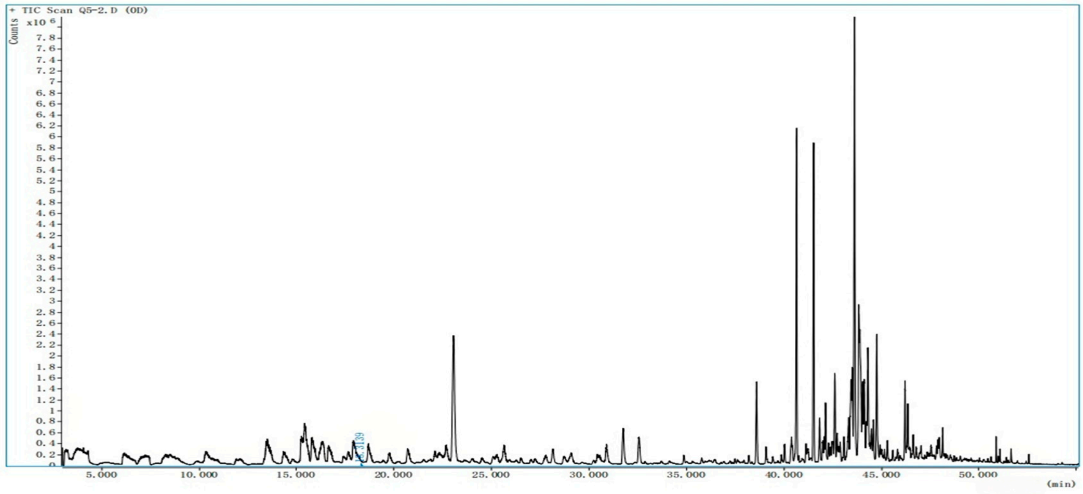
+ TIC Scan Q5-2.D (OD)
Counts
x10 6
(min)

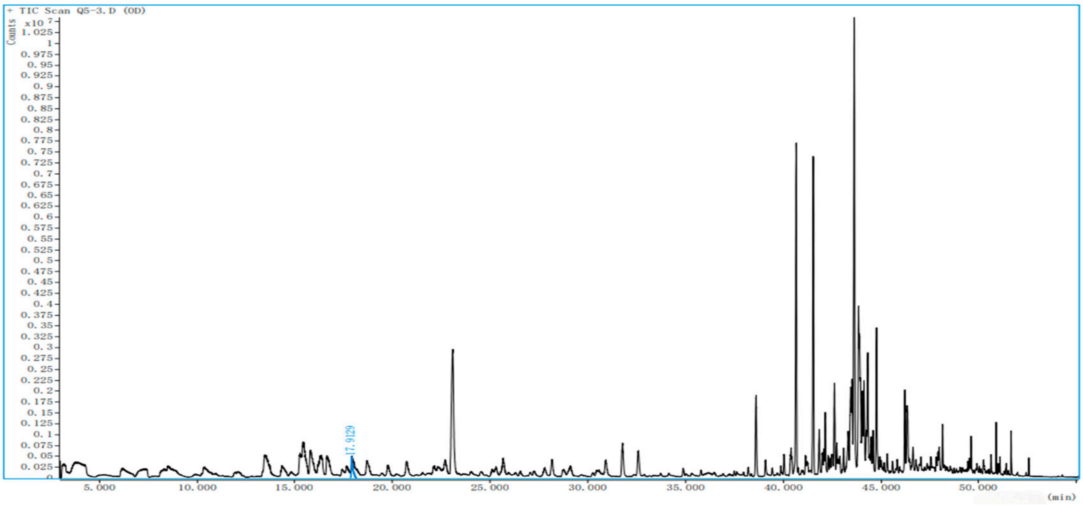
+ TIC Scan Q5-3.D (OD)
Counts
x10 7
17.9129
(min)

(B) Q6

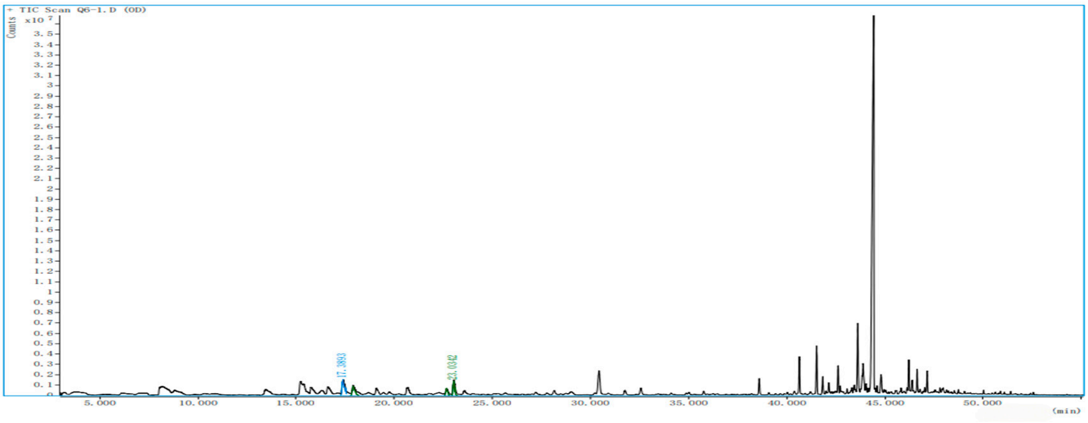

TIC Scan Q6-1.D (OD)
Counts
x10 7
(min)

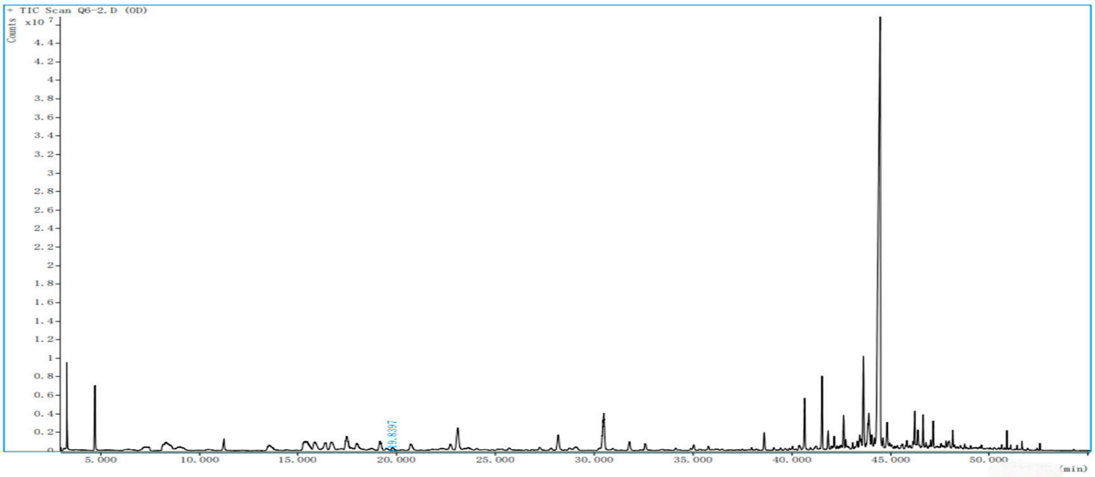

TIC Scan Q6-2.D (OD)
Counts
x10 7
(min)

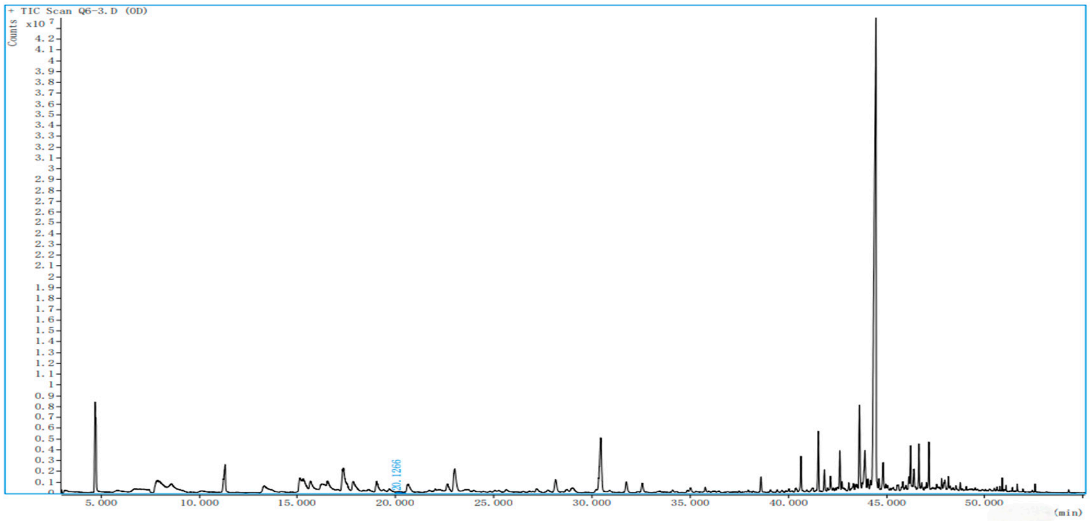

TIC Scan Q6-3.D (OD)
Counts
x10 7
(min)

(C) Q7

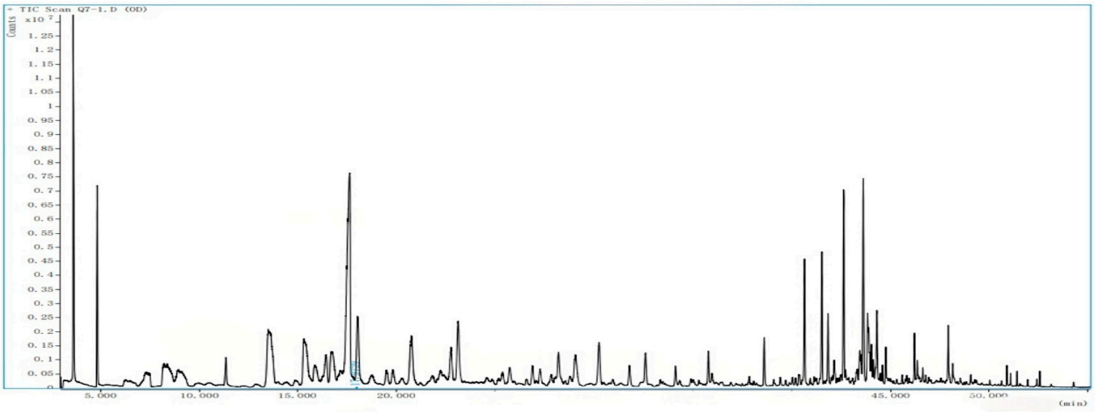
+ TIC Scan Q7-1.D (OD)
Counts
x10 7
(min)

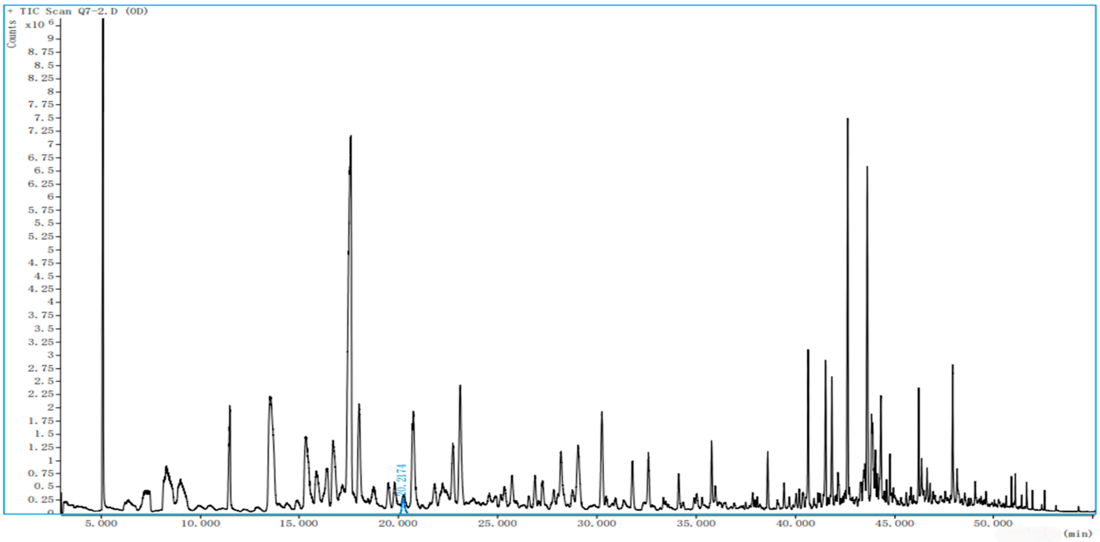
+ TIC Scan Q7-2.D (OD)
Counts
x10 6
(min)

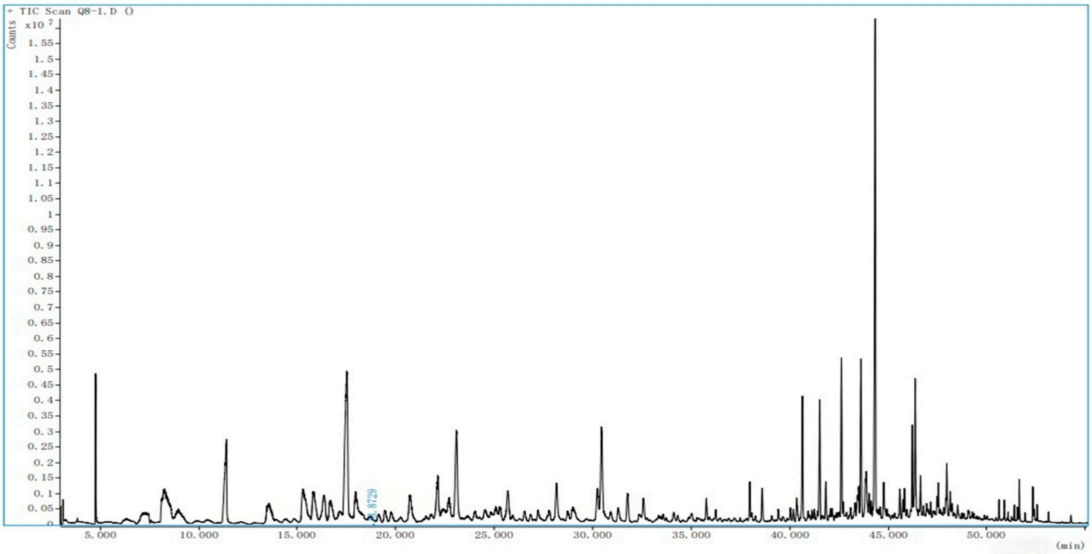
+ TIC Scan Q8-1.D ()
Counts
x10 7
(min)

(D) Q8

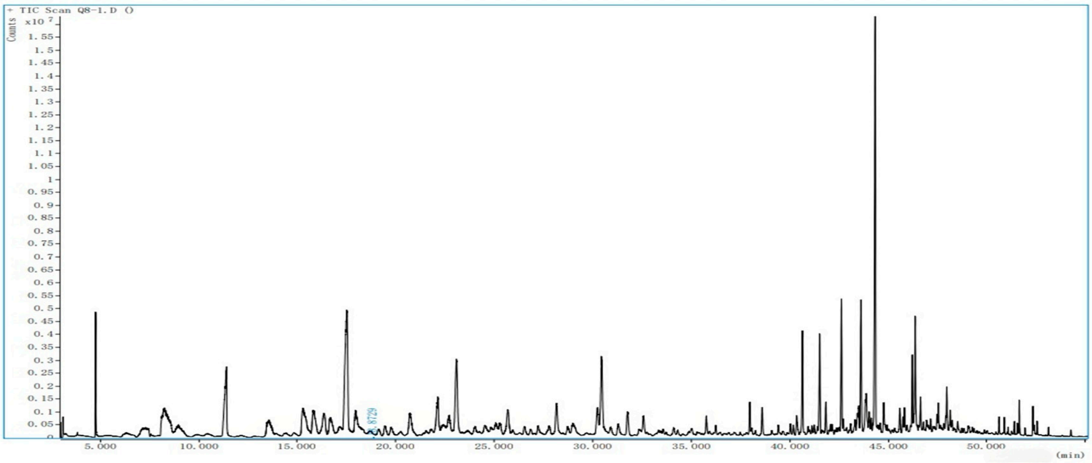
+ TIC Scan Q8-1.D ()
Counts
x10 7
(min)

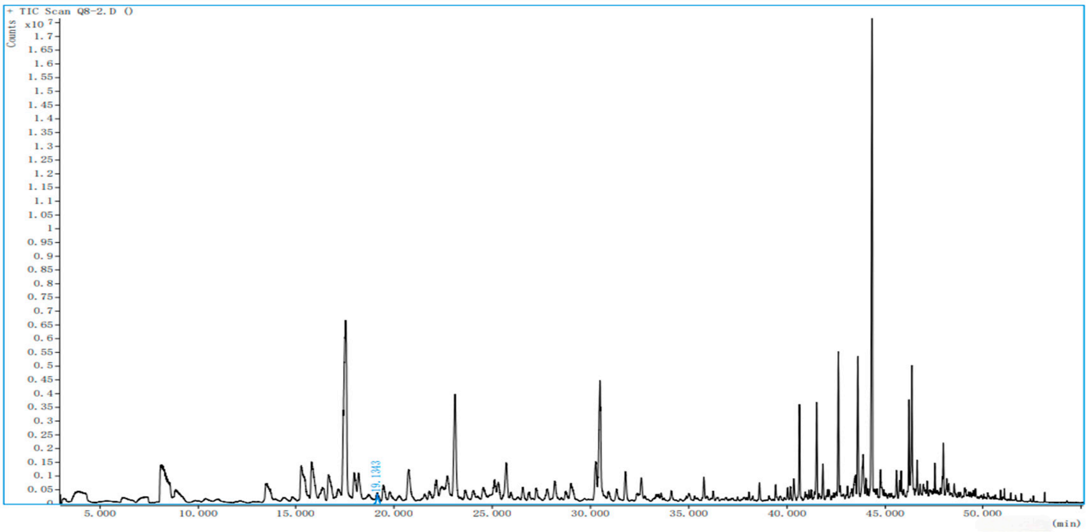
+ TIC Scan Q8-2.D ()
Counts
x10 7
19.1343
(min)

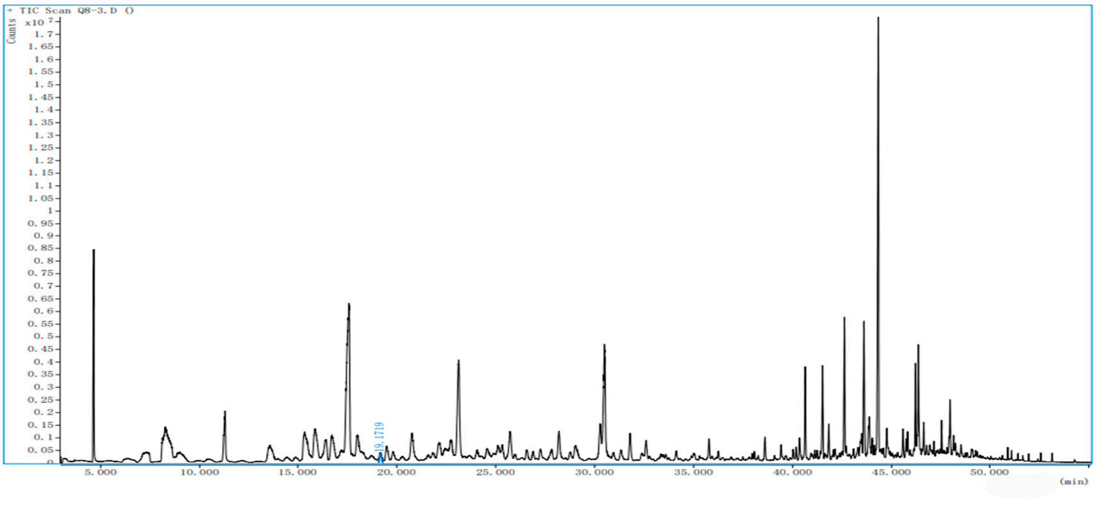
+ TIC Scan Q8-3.D ()
Counts
x10 7
19.1719
(min)

(E) Q9

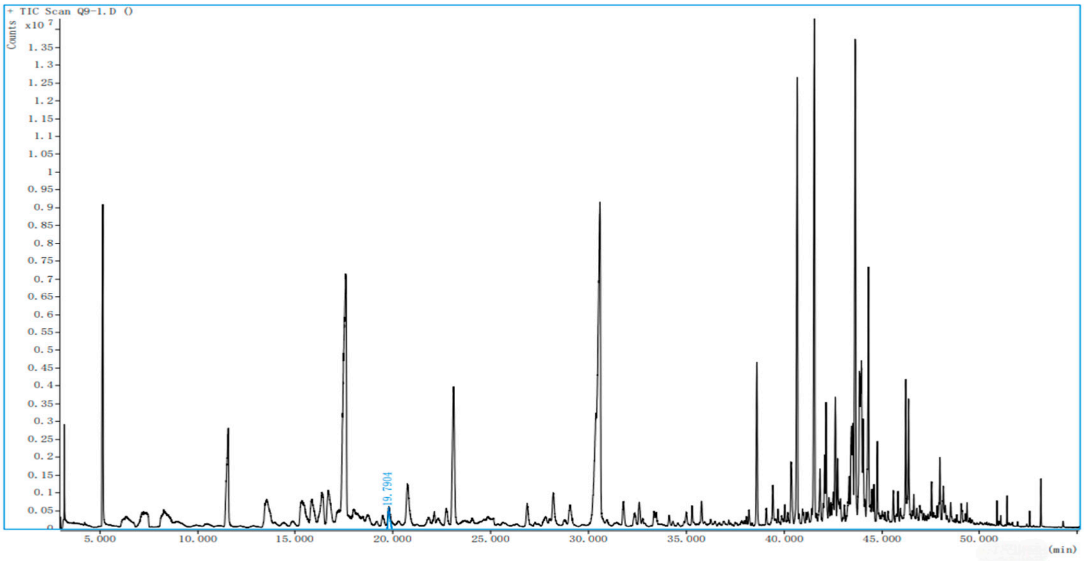
+ TIC Scan Q9-1.D ()
Counts x10 7
19.7904
(min)

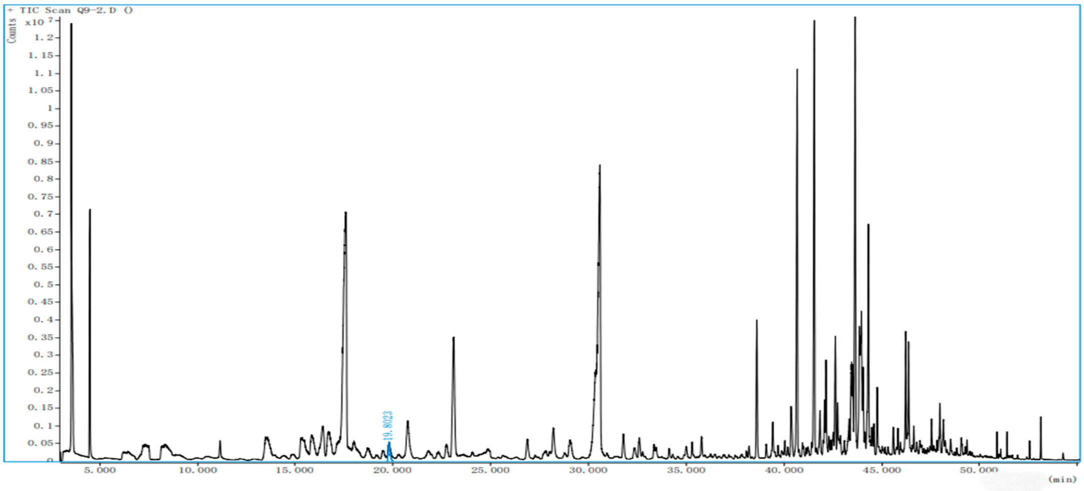
+ TIC Scan Q9-2.D ()
Counts x10 7
19.8023
(min)

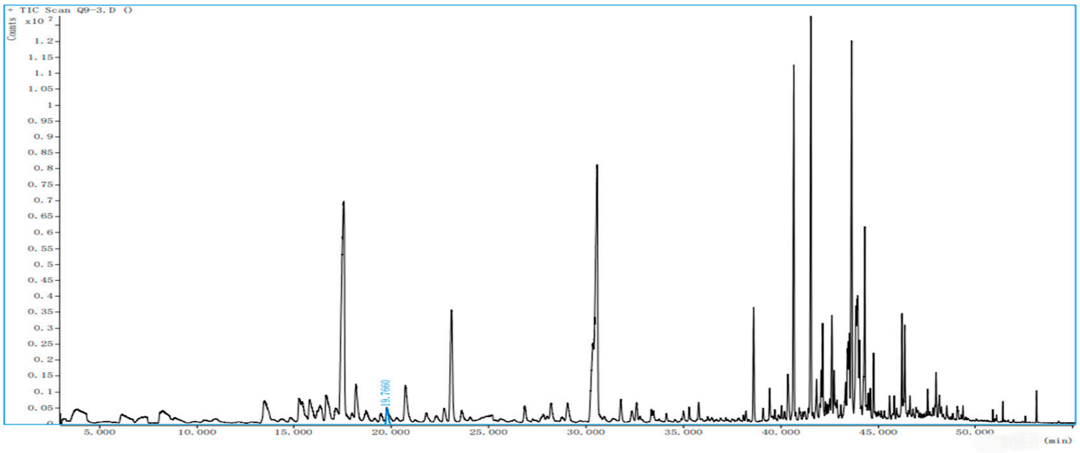
+ TIC Scan Q9-3.D ()
Counts x10 7
19.7660
(min)

Figure S2. Total ion chromatograms (TICs) obtained by SPME–GC–MS analysis of *C. paliurus* leaf powder samples collected from May to September. Note: Total ion chromatograms of three independent biological replicates are shown for each harvest month: (A) Q5, May; (B) Q6, June; (C) Q7, July; (D) Q8, August; and (E) Q9, September. The chromatograms were obtained from oven-dried *C. paliurus* leaf powder samples analyzed by solid-phase microextraction coupled with gas chromatography–mass spectrometry (SPME–GC–MS). The x-axis represents retention time in minutes, and the y-axis represents ion intensity. VOC annotations were considered tentative and were screened based on mass spectral matching and retention index comparison. Corresponding retention times, retention indices, and peak-area data are provided in Supplementary Table S5.

Supplementary Table S

Table S1. Monthly meteorological conditions and extraction yields at Shimen (Hunan, China) during the sampling period (May–September 2024).

Sampling time point	Sampling date	Rainfall (mm)	Rainy days (d)	Max temp (°C)	Mean temp (°C)	Min temp (°C)	Extraction yield (%)
Q5	3 May 2024	173.12	8	25	20	16	18.2 ± 0.6
Q6	1 Jun 2024	462.60	11	26	22	19	19.5 ± 0.7
Q7	3 Jul 2024	123.47	1	34	26	21	17.8 ± 0.5
Q8	6 Aug 2024	134.21	1	33	26	20	20.1 ± 0.8
Q9	1 Sep 2024	189.16	5	29	24	19	19.0 ± 0.6

Note: Meteorological data were obtained from the WorldWeatherOnline platform for Shimen, Hunan, China, and summarized for the sampling months (May－September 2024).

Supplementary Table S2. Tentatively annotated volatile organic compounds in *C. paliurus* leaves harvested at different developmental stages (Q5 – Q9), with retention index verification.

Class	Compound name	CAS No.	Q5					Q6					Q7					Q8					Q9				
			Content(%)	RT (min)	Calc. RI	Ref. RI	RSD (%)	Content(%)	RT (min)	Calc. RI	Ref. RI	RSD (%)	Content(%)	RT (min)	Calc. RI	Ref. RI	RSD (%)	Content(%)	RT (min)	Calc. RI	Ref. RI	RSD (%)	Content(%)	RT (min)	Calc. RI	Ref. RI	RSD (%)
Alkenes/terpenes	7-epi-Silphiperfol-5-ene	138752-23-5	18.51 ± 10.31	43.692	1356.29	1348.00	55.71	6.07 ± 0.61	43.754	1352.25	1348.00	10.13	4.69 ± 2.66	43.872	1356.49	1348.00	56.73	5.95 ± 5.45	43.720	1352.25	1348.00	91.60	19.28 ± 7.88	43.704	1352.66	1348.00	40.89
	α-Cubebene	17699-14-8	9.07 ± 1.22	43.505	1350.21	1351.00	13.50	1.05 ± 0.25	43.441	1349.20	1351.00	23.91	2.50 ± 1.87	43.447	1349.31	1351.00	74.86	2.54 ± 0.90	43.478	1350.06	1351.00	35.55	7.43 ± 1.16	43.494	1350.49	1351.00	15.64
	δ-Elemene	20307-84-0	2.10 ± 0.29	41.830	1319.36	1338.00	13.99	—	—	—	—	—	—	—	—	—	—	—	—	—	—	—	1.93 ± 0.17	41.838	1319.55	1338.00	9.00
	(+)-Cyclosativene	22469-52-9	9.70 ± 5.59	44.405	1365.08	1368.00	57.57	—	—	—	—	—	0.70 ± 0.05	44.593	1370.35	1368.00	6.75	—	—	—	—	—	2.21 ± 1.46	44.561	1370.35	1368.00	66.02
	dl-Neoisolongifolene	26783-22-2	1.68 ± 0.88	43.007	1346.82	1330.00	52.69	—	—	—	—	—	—	—	—	—	—	—	—	—	—	—	—	—	—	—	—
	α-Guaiene	3691-12-1	1.33 ± 0.58	47.924	1436.69	1439.00	43.69	—	—	—	—	—	—	—	—	—	—	—	—	—	—	—	—	—	—	—	—
	α-Copaene	3856-25-5	0.37 ± 0.22	45.011	1378.00	1376.00	59.50	—	—	—	—	—	—	—	—	—	—	—	—	—	—	—	—	—	—	—	—
	α-Fenchene	471-84-1	1.61 ± 0.38	14.365	945.19	950.00	23.72	—	—	—	—	—	—	—	—	—	—	—	—	—	—	—	—	—	—	—	—
	pseudo-Limonene	499-97-8	0.70 ± 0.11	17.649	997.59	1004.00	15.27	—	—	—	—	—	—	—	—	—	—	—	—	—	—	—	—	—	—	—	—
	2,3-Dimethyl-1,3-butadie	513-81-5	1.99 ± 0.34	3.186	606.61	611.00	17.11	—	—	—	—	—	—	—	—	—	—	—	—	—	—	—	—	—	—	—	—

ne																										
β-Elemene	515-13-9	0.59 ± 0.14	45.827	1393.04	1391.00	23.17	—	—	—	—	—	—	—	—	—	—	1.97 ± 0.30	45.817	1392.80	1391.00	15.25	1.45 ± 0.44	45.741	1388.50	1391.00	29.99
α-Longipinene	5989-08-2	3.69 ± 1.83	43.602	1360.00	1353.00	49.47	18.10 ± 26.92	44.120	1357.43	1353.00	148.74	—	—	—	—	—	—	—	—	—	—	—	—	—	—	—
2-Isobutyl-6-methyl-1-heptene	7323-15-1	2.00 ± 0.26	25.662	1109.54	1119.00	13.17	—	—	—	—	—	1.76 ± 0.31	26.862	1123.48	1119.00	17.64	0.77 ± 0.29	26.248	1123.58	1119.00	37.19	—	—	—	—	—
1-Decene	872-05-9	2.50 ± 0.62	17.919	1001.53	989.00	24.88	—	—	—	—	—	—	—	—	—	—	—	—	—	—	—	—	—	—	—	—
α-Ylangene	14912-44-8	7.21 ± 1.10	44.761	1373.37	1372.00	15.25	47.42 ± 39.70	44.574	1367.88	1372.00	83.72	1.67 ± 0.31	44.760	1373.29	1372.00	18.62	4.10 ± 0.39	44.773	1373.62	1372.00	9.57	—	—	—	—	—
β-Longipinene	41432-70-6	2.39 ± 0.42	46.349	1395.32	1403.00	17.58	2.16 ± 0.85	46.473	1404.08	1403.00	39.36	—	—	—	—	—	9.09 ± 0.90	46.363	1403.47	1403.00	9.91	3.98 ± 0.40	46.362	1403.38	1403.00	10.18
Thujopsene	470-40-6	0.70 ± 0.33	47.435	1418.10	1429.00	47.38	—	—	—	—	—	—	—	—	—	—	1.58 ± 0.50	46.740	1409.26	1429.00	31.42	0.67 ± 0.18	46.633	1409.24	1429.00	26.50
β-Patchoulene	514-51-2	0.40 ± 0.16	45.154	1380.62	1381.00	40.68	—	—	—	—	—	—	—	—	—	—	—	—	—	—	—	—	—	—	—	—
β-Caryophyllene	87-44-5	—	—	—	—	—	1.84 ± 0.63	47.151	1420.49	1419.00	34.14	—	—	—	—	—	1.08 ± 0.67	47.344	1428.86	1419.00	62.03	—	—	—	—	—
Panaxene	871660-95-6	—	—	—	—	—	5.78 ± 3.56	41.229	1298.17	1314.00	61.53	6.29 ± 1.65	41.227	1313.82	1314.00	26.23	6.65 ± 0.72	40.933	1298.17	1314.00	10.84	18.76 ± 3.74	41.254	1314.32	1314.00	19.95
Isocaryophyllene	118-65-0	—	—	—	—	—	2.06 ± 0.36	46.644	1409.54	1406.00	17.23	—	—	—	—	—	—	—	—	—	—	—	—	—	—	—
Silphinene	74284-57-4	—	—	—	—	—	6.27 ± 1.71	43.616	1352.24	1346.00	27.30	—	—	—	—	—	—	—	—	—	—	—	—	—	—	—

	α-Terpinene	99-86-5	—	—	—	—	—	1.28 ± 0.42	19.120	1018.59	1017.00	33.18	—	—	—	—	—	—	—	—	—	—	—	—	—	—	—
	(+)-Aromadendrene	489-39-4	—	—	—	—	—	—	—	—	—	—	3.56 ± 0.51	47.919	1437.12	1440.00	14.44	—	—	—	—	—	—	—	—	—	—
	(Z)-2-Undecene	821-96-5	—	—	—	—	—	—	—	—	—	—	2.15 ± 0.05	25.716	1110.49	1102.00	2.48	3.26 ± 1.25	25.486	1109.96	1102.00	38.34	—	—	—	—	—
	3-carene	13466-78-9	—	—	—	—	—	—	—	—	—	—	—	—	—	—	—	1.13 ± 0.13	19.154	1019.65	1011.00	11.85	—	—	—	—	—
	trans-Pinane	33626-25-4	—	—	—	—	—	—	—	—	—	—	—	—	—	—	—	1.49 ± 0.05	17.169	989.66	988.00	3.34	—	—	—	—	—
	Longifolene	475-20-7	—	—	—	—	—	—	—	—	—	—	—	—	—	—	—	1.92 ± 0.18	46.107	1409.26	1405.00	9.40	—	—	—	—	—
	8-Methyl-1-decene	61142-79-8	—	—	—	—	—	—	—	—	—	—	—	—	—	—	—	1.98 ± 0.35	22.291	1062.07	1056.00	17.58	—	—	—	—	—
	Cascarilladiene	59742-39-1	—	—	—	—	—	—	—	—	—	—	—	—	—	—	—	—	—	—	—	—	2.77 ± 0.31	44.764	1373.55	1372.00	11.08
	cis-Carveol	1197-06-4	0.28 ± 0.02	35.785	1230.06	1229.00	6.74	—	—	—	—	—	2.19 ± 0.04	35.772	1230.01	1229.00	1.82	1.67 ± 0.17	35.772	1230.08	1229.00	9.89	1.00 ± 0.13	35.780	1230.15	1229.00	12.61
	cis-Sabinene hydrate	15537-55-0	1.15 ± 0.17	22.698	1069.88	1070.00	14.72	1.03 ± 0.22	22.645	1069.55	1070.00	20.82	—	—	—	—	—	—	—	—	—	—	1.24 ± 0.18	22.730	1070.41	1070.00	14.11
Alcohols	Thujyl alcohol	513-23-5	0.26 ± 0.03	30.245	1161.60	1168.00	11.66	—	—	—	—	—	—	—	—	—	—	0.80 ± 0.04	30.925	1169.24	1168.00	4.99	—	—	—	—	—
	(-)-trans-Pinocarveol	547-61-5	1.02 ± 0.08	27.786	1133.80	1139.00	7.67	—	—	—	—	—	—	—	—	—	—	—	—	—	—	—	—	—	—	—	—
	4-Hepten-1-ol	20851-55-2	1.37 ± 0.12	10.361	873.81	870.00	8.83	—	—	—	—	—	—	—	—	—	—	—	—	—	—	—	—	—	—	—	—

Dihydroterpineol	21129-27-1	3.04 ± 0.15	31.752	1178.80	1178.00	4.83	—	—	—	—	—	—	—	—	—	—	—	—	—	—	—	—	—	—	—	—
trans-2-Nonen-1-ol	31502-14-4	—	—	—	—	—	1.02 ± 0.43	31.758	1178.65	1176.00	42.35	—	—	—	—	—	1.51 ± 0.05	31.320	1173.77	1176.00	3.57	1.65 ± 0.06	31.788	1179.23	1176.00	3.43
3,3,5-Trimethylcyclohexanol	116-02-9	—	—	—	—	—	—	—	—	—	—	9.12 ± 0.25	23.096	1075.52	1073.00	2.77	—	—	—	—	—	—	—	—	—	—
6-Methyl-5-hepten-2-ol	1569-60-4	—	—	—	—	—	—	—	—	—	—	1.12 ± 0.73	17.142	989.66	994.00	64.95	—	—	—	—	—	—	—	—	—	—
trans-Sabinene hydrate	17699-16-0	—	—	—	—	—	—	—	—	—	—	4.54 ± 1.14	22.734	1070.81	1070.00	25.12	—	—	—	—	—	—	—	—	—	—
Thujyl alcohol	21653-20-3	—	—	—	—	—	—	—	—	—	—	0.63 ± 0.11	31.347	1174.24	1176.00	17.74	—	—	—	—	—	—	—	—	—	—
4-Methyl-1-pentanol	626-89-1	—	—	—	—	—	—	—	—	—	—	4.08 ± 1.05	9.071	845.34	846.00	25.72	—	—	—	—	—	—	—	—	—	—
trans-Isopiperitenol	74410-00-7	—	—	—	—	—	—	—	—	—	—	1.71 ± 0.08	34.110	1206.69	1210.00	4.39	—	—	—	—	—	0.53 ± 0.06	34.117	1206.89	1210.00	10.67
cis-Ocimenol	7643-59-6	—	—	—	—	—	—	—	—	—	—	5.62 ± 0.67	30.240	1161.67	1157.00	11.95	3.64 ± 1.06	30.248	1161.87	1157.00	29.17	—	—	—	—	—
(-)-Isopulegol	89-79-2	—	—	—	—	—	—	—	—	—	—	5.67 ± 0.37	29.042	1148.11	1146.00	6.53	—	—	—	—	—	1.99 ± 0.10	29.040	1148.05	1146.00	5.10
2-Cyclohexen-1-ol,2-methyl-5-(1-methylethenyl)-,(1R,5S)-rel-	1197-07-5	—	—	—	—	—	—	—	—	—	—	—	—	—	—	—	1.01 ± 0.17	34.106	1206.76	1217.00	16.71	—	—	—	—	—

	Ethanol,2-(3,3-dimethylcyclohexylidene)-, (2Z)-	26532-23-0	—	—	—	—	—	—	—	—	—	—	—	—	—	—	—	0.83 ± 0.41	35.491	1219.02	1225.00	48.95	—	—	—	—	—
	(Z)-p-Mentha-2,8-dien-1-ol	3886-78-0	—	—	—	—	—	—	—	—	—	—	—	—	—	—	—	0.50 ± 0.02	23.654	1083.58	1102.00	3.09	—	—	—	—	—
	cis-Verbenol	13040-03-4	—	—	—	—	—	—	—	—	—	—	—	—	—	—	—	—	—	—	—	—	0.44 ± 0.06	28.791	1145.40	1140.00	14.39
	δ-Terpineol	7299-42-5	—	—	—	—	—	—	—	—	—	—	—	—	—	—	—	—	—	—	—	—	6.90 ± 1.50	30.358	1162.96	1166.00	21.77
	iso-3-Thujanol	7712-79-0	—	—	—	—	—	—	—	—	—	—	—	—	—	—	—	—	—	—	—	—	0.68 ± 0.09	27.809	1134.03	1136.00	13.38
	6-Methyl-6-hepten-2-one	10408-15-8	2.39 ± 1.32	15.368	961.67	966.00	55.11	2.20 ± 1.77	15.231	964.80	966.00	80.33	10.80 ± 1.90	15.292	960.01	966.00	17.59	8.37 ± 0.81	15.291	959.41	966.00	9.71	2.08 ± 0.13	15.338	960.38	966.00	6.34
Ketones	β-Damascone	35044-68-9	1.04 ± 0.29	46.627	1409.13	1417.00	28.07	—	—	—	—	—	—	—	—	—	—	—	—	—	—	—	—	—	—	—	—
	Umbellulone	24545-81-1	1.57 ± 0.12	30.931	1169.35	1171.00	7.46	—	—	—	—	—	—	—	—	—	—	—	—	—	—	—	—	—	—	—	—
	Dihydrojasmone	1128-08-1	—	—	—	—	—	—	—	—	—	—	0.42 ± 0.01	44.936	1376.68	1369.00	1.49	—	—	—	—	—	—	—	—	—	—

	cis-Arbusculone	56469-37-5	—	—	—	—	—	—	—	—	—	—	1.59 ± 0.22	21.798	1057.49	1052.00	13.66	—	—	—	—	—	—	—	—	—	—
	tridecan-2-one	593-08-8	—	—	—	—	—	—	—	—	—	—	0.53 ± 0.06	51.078	1506.11	1497.00	11.02	—	—	—	—	—	—	—	—	—	—
	2-methyl-2-pentenal	623-36-9	—	—	—	—	—	—	—	—	—	—	5.36 ± 5.00	8.261	828.30	837.00	93.16	—	—	—	—	—	—	—	—	—	—
	Hexanal	66-25-1	—	—	—	—	—	—	—	—	—	—	1.97 ± 0.57	6.264	780.52	800.00	28.90	—	—	—	—	—	—	—	—	—	—
	2-ethylhexanal	123-05-7	—	—	—	—	—	—	—	—	—	—	—	—	—	—	—	0.54 ± 0.10	14.833	952.05	956.00	17.71	0.59 ± 0.07	14.859	953.59	956.00	11.54
Aldeh ydes	(Z)-6-Nonenal	2277-19-2	—	—	—	—	—	—	—	—	—	—	—	—	—	—	—	1.12 ± 0.08	24.045	1089.50	1101.00	6.73	—	—	—	—	—
	(E)-2-ethylbut-2-enal	63883-69-2	—	—	—	—	—	—	—	—	—	—	—	—	—	—	—	6.76 ± 8.07	8.188	829.18	819.00	119.39	—	—	—	—	—
	10-Undecenal	112-45-8	—	—	—	—	—	—	—	—	—	—	—	—	—	—	—	—	—	—	—	—	0.41 ± 0.04	39.689	1284.81	1288.00	9.31
Esters	2-Cyclohexen-1-ol,	1204-30	—	—	—	—	—	1.35 ±	41.835	1319.64	1303.00	11.93	4.01 ±	41.835	1319.57	1303.00	2.67	2.31 ±	41.83	1319.57	1303.00	6.28	—	—	—	—	—

	3-methyl-6-(1-methylethyl)-, acetate	-4						0.16					0.11					0.14	3								
	7,7-Dimethylbicyclo[2.2.1]hept-2-yl acetate	62555-02-6	—	—	—	—	—	—	—	—	—	—	—	—	—	—	—	0.43 ± 0.05	32.738	1198.09	1181.00	11.82	—	—	—	—	—
	neoiso-3-thujyl acetate	62181-91-3	—	—	—	—	—	—	—	—	—	—	—	—	—	—	—	—	—	—	—	—	5.33 ± 0.38	38.602	1269.64	1280.00	7.06
Ethers/furans	2,3-Dihydro-2,2-dimethyl-7-benzofuranol	1563-38-8	0.36 ± 0.09	39.428	1281.06	1286.00	23.87	—	—	—	—	—	0.73 ± 0.21	39.421	1281.09	1286.00	29.28	—	—	—	—	—	1.59 ± 0.33	39.421	1281.08	1286.00	20.65
	Dill ether	74410-10-9	2.37 ± 0.29	32.572	1187.89	1186.00	12.40	0.89 ± 0.11	32.571	1187.85	1186.00	12.45	2.60 ± 0.02	32.582	1188.22	1186.00	0.76	1.85 ± 0.06	32.589	1188.37	1186.00	3.17	1.22 ± 0.08	32.590	1188.32	1186.00	6.35
Alkanes	Octylcyclopropane	1472-09-9	0.52 ± 0.06	24.547	1096.44	1095.00	10.79	—	—	—	—	—	—	—	—	—	—	11.16 ± 8.50	23.578	1075.98	1095.00	76.19	—	—	—	—	—
	tert-Pentylcyclohexane	31797-64-5	—	—	—	—	—	—	—	—	—	—	1.62 ± 0.11	27.242	1127.73	1130.00	6.89	—	—	—	—	—	—	—	—	—	—

	1-Methyl-2-(1-methylpentyl)cyclopropane	62238-06-6	—	—	—	—	—	—	—	—	—	—	0.68 ± 0.03	14.402	945.94	947.00	4.07	—	—	—	—	—	—	—	—	—	—
	3,9-dimethyl undecane	17301-31-4	—	—	—	—	—	—	—	—	—	—	—	—	—	—	—	0.69 ± 0.05	36.248	1236.63	1240.00	7.46	—	—	—	—	—
	Cyclodecane	293-96-9	—	—	—	—	—	—	—	—	—	—	—	—	—	—	—	3.30 ± 0.37	29.020	1148.05	1147.00	11.27	—	—	—	—	—
	Cyclooctane, 1,4-dimethyl-, cis-	13151-99-0	—	—	—	—	—	—	—	—	—	—	—	—	—	—	—	—	—	—	—	—	4.39 ± 0.21	20.737	1041.94	1054.00	4.86
	Tridecane	629-50-5	—	—	—	—	—	—	—	—	—	—	—	—	—	—	—	—	—	—	—	—	0.44 ± 0.02	40.941	1303.09	1300.00	4.11
Alkynes	1-Undecyne	2243-98-3	15.52 ± 1.48	23.074	1075.45	1095.00	9.53	—	—	—	—	—	—	—	—	—	—	7.42 ± 5.74	27.425	1075.45	1095.00	77.42	11.05 ± 1.05	23.098	1075.72	1095.00	9.51
	dec-2-yne	2384-70-5	1.79 ± 0.17	20.706	1041.28	1041.00	9.48	1.49 ± 0.16	20.676	1040.57	1041.00	10.73	9.28 ± 0.67	20.734	1042.21	1041.00	7.18	—	—	—	—	—	—	—	—	—	—

Aromatic hydrocarbons	2-(1,3-Butadienyl)mesitylene	5732-00-3	0.81 ± 0.06	45.302	1383.32	1373.00	7.74	—	—	—	—	—	—	—	—	—	—	—	—	—	—	—	—	—	—	—	—
	Benzene,(3-methylbutyl)-	2049-94-7	—	—	—	—	—	—	—	—	—	—	0.76 ± 0.27	23.675	1082.92	1098.00	36.32	—	—	—	—	—	—	—	—	—	—
Nitrogen-containing compounds	Emylcamate	78-28-4	—	—	—	—	—	—	—	—	—	—	1.06 ± 0.65	24.571	1096.91	1105.00	61.65	—	—	—	—	—	—	—	—	—	—
Other oxygenated compounds	Ascaridole	512-85-6	—	—	—	—	—	—	—	—	—	—	0.60 ± 0.08	37.844	1258.99	1244.00	12.85	—	—	—	—	—	—	—	—	—	—
	Edulan I	41678-29-9	—	—	—	—	—	—	—	—	—	—	—	—	—	—	—	2.60 ± 3.78	41.441	1313.64	1314.00	145.32	—	—	—	—	—

Notes: RT, retention time; RI, retention index; RSD, relative standard deviation. Q5–Q9 represent samples harvested from May to September, respectively. Content values are expressed as mean ± SD (%) based on normalized peak areas from three sampling-block replicates. Only compounds detected in at least two of the three replicates were retained for each harvest month. Volatile compounds were tentatively annotated based on mass spectral information and RI verification. Calc. RI

values refer to the RI values generated in the original GC–MS data-processing output, whereas Ref. RI values were obtained from library/reference records associated with the tentative annotations. RI deviation was calculated as |Calc. RI − Ref. RI|. Compounds with unreliable annotation or an RI deviation greater than 30 were excluded from the final annotated VOC list. No mass spectral matching score was used as a retention criterion in the revised table. Compounds with relatively high RSD values were retained only for qualitative profiling and trend description and should not be interpreted as robust quantitative markers.

Table S3. Supplementary dataset used to generate Figure 2A: concentration-dependent DPPH radical scavenging activities of aqueous extracts from *C. paliurus* leaves harvested in different months.

Concentration (mg/mL)	Q5	Q5-SD	Q6	Q6-SD	Q7	Q7-SD	Q8	Q8-SD	Q9	Q9-SD	VC	VC-SD
0.2	1.7523	3.7981	0.8178	1.7288	6.8925	2.2562	1.9081	6.1087	20.6776	2.3277	98.756	0.22
0.5	3.3879	1.4915	17.1729	0.2023	9.3069	3.096	6.6978	5.4973	25.1947	2.8623	98.4889	0.22
1	5.4907	0.7106	30.1791	1.6201	7.9439	0.5092	5.3738	2.8037	44.2757	5.1917	99.1689	0.22
2	14.0966	0.3569	54.2056	3.1369	17.095	0.7778	10.8645	1.7209	69.9766	1.2197	98.8289	0.22
3.5	23.3255	0.7952	77.9206	3.0974	30.6075	9.7216	22.1573	7.4201	84.4237	0.0674	99.83	0.22
5	34.9688	1.1346	86.176	1.2921	30.6854	1.7341	19.3925	3.6422	84.7352	1.0471	98.8289	0.22

Note: Note: Values are presented as mean ± standard deviation (SD) of three biological replicates (n = 3). Q5 – Q9 denote *C. paliurus* leaf samples collected from May to September, respectively. VC denotes vitamin C used as the positive control. The listed concentrations represent the final extract concentrations in the reaction mixture. These data were used to generate the DPPH radical scavenging curves shown in Figure 2A. DPPH, 2,2-diphenyl-1-picrylhydrazyl.

Table S4. Individual raw fluorescence intensity values of advanced glycation end-products (AGEs) formation in the Glu–BSA and Fru–BSA models used for the anti-glycation evaluation of *C. paliurus* leaf extracts.

	Week	Q5			Q6			Q7			Q8			Q9			Control			AG		
Glu	0	12744	15610	16018	10959	13758	13928	15058	17712	18192	15314	19293	19219	16198	20056	20993	17245	22345	22267	0.00	0.00	0.00
	1	24007	35808	28176	11111	13528	14304	25456	28738	28154	28511	35171	36159	11741	14192	14477	20453	25838	23131	13155	13422	13688
	2	10029	12416	12200	7823	8352	7514	11603	13676	13728	13772	14303	14766	6504	7080	7998	30633	28415	28811	9026	9372	9717
	3	11684	13568	14121	7110	8288	8447	12832	14744	14770	12890	15340	15505	6250	7004	7372	32356	31813	31857	7313	7682	8051
	4	13002	13839	13464	7755	7888	8516	14038	16340	16063	14332	16460	16226	6968	7153	7082	50790	48515	45811	6143	6772	7401
Fru	0	9042	9848	11988	6463	6597	7384	13780	10062	10689	14740	14241	14789	15369	18828	17281	20261	20674	18498	0.00	0.00	0.00
	1	7926	9186	8992	5545	6302	6960	8070	8104	10540	8606	10600	11012	5539	6639	6837	29060	27118	29568	8791	9120	9449
	2	32541	38221	39222	32541	38221	39222	32387	38205	42939	35019	42489	45913	13321	16030	16903	39704	43085	38563	10849	11340	11831
	3	28335	37308	39826	14751	18438	18919	33338	38716	41674	34062	43686	44894	13071	15899	15412	39380	50576	52859	9880	10450	11020
	4	36119	34772	34857	20447	18873	18880	32947	39081	41363	34215	43670	41717	13441	15505	15499	51138	52124	48636	7474	8080	8686

Note: Glu – BSA and Fru – BSA denote glucose – bovine serum albumin and fructose – bovine serum albumin glycation models, respectively. Q5 – Q9 represent *C. paliurus* leaf extracts prepared from leaves collected from May to September, respectively. Control denotes the negative control group receiving methanol instead of leaf extract or aminoguanidine hydrochloride. AG denotes aminoguanidine hydrochloride used as the positive reference inhibitor. Incubation time is expressed in weeks. Data are raw fluorescence intensity values of AGEs expressed as relative fluorescence units (RFU), measured at excitation/emission wavelengths of 330/410 nm.

Table S5. Summary of volatile organic compounds (VOCs) detected in *C. paliurus* leaves that appeared in at least two harvest months, including retention indices (RI) and peak areas.

CAS	Formula	Name	RI_theoretical	RI	Q5-1_RT	Q5-1_Area	Q5-2_RT	Q5-2_Area	Q5-3_RT	Q5-3_Area
10408-15-8	$C_8H_{14}O$	6-Methyl-6-hepten-2-one	961.6741501	966	15.406	2,184,841.42	15.439	5,272,204.37	15.260	2,208,916.71
1197-06-4	$C_{10}H_{16}O$	cis-Carveol	1230.062981	1229	35.776	348,703.74	35.785	387,055.71	35.794	396,819.68
138752-23-5	$C_{15}H_{24}$	7-epi-Silphiperfol-5-ene	1356.285662	1348	43.831	9,213,007.81	43.625	35,553,403.52	43.620	30,125,021.97
15537-55-0	$C_{10}H_{18}O$	cis-Sabinene hydrate	1069.877108	1070	22.686	1,311,691.82	22.695	1,766,390.85	22.713	1,583,723.71
1563-38-8	$C_{10}H_{12}O_2$	2,3-Dihydro-2,2-dimethyl-7-benzofuranol	1281.063681	1286	39.420	353,167.15	39.430	525,773.33	39.434	572,333.11
17699-14-8	$C_{15}H_{24}$	α-Cubebene	1350.211669	1351	43.501	11,186,351.13	43.510	14,130,977.76	43.505	11,366,698.16
20307-84-0	$C_{15}H_{24}$	δ-Elemene	1319.363151	1338	41.825	2,453,983.56	41.830	3,243,838.39	41.834	2,796,805.27
22469-52-9	$C_{15}H_{24}$	(+)-Cyclosativene	1365.083747	1368	44.309	14,387,959.14	44.314	19,887,442.46	44.593	4,986,700.51
26783-22-2	$C_{15}H_{24}$	dl-Neoisolongifolene	1346.824959	1330	43.317	3,093,951.45	42.386	896,302.49	43.317	2,788,903.33
35044-68-9	$C_{13}H_{20}O$	β-Damascone	1409.128361	1417	46.627	1,070,268.18	46.628	1,289,706.47	46.626	1,832,273.27
3691-12-1	$C_{15}H_{24}$	α-Guaiene	1436.686904	1439	47.898	960,725.26	47.913	1,896,711.20	47.962	2,514,752.32
3856-25-5	$C_{15}H_{24}$	α-Copaene	1378.004786	1376	45.011	340,073.11	45.012	844,892.98	45.011	317,875.38

471-84-1	$C_{10}H_{16}$	α-Fenchene	945.1892239	950	14.378	1,606,579.97	14.333	2,617,120.92	14.383	2,273,444.40
499-97-8	$C_{10}H_{16}$	pseudo-Limonene	997.5946119	1004	17.646	1,026,054.77	17.646	1,014,982.26	17.656	772,623.15
513-23-5	$C_{10}H_{18}O$	Thujyl alcohol	1161.602898	1168	30.240	304,123.63	30.236	375,651.49	30.259	374,510.81
513-81-5	C_6H_{10}	2,3-Dimethyl-1,3-butadiene	606.6071429	611	3.171	3,202,267.88	3.198	2,478,748.95	3.189	2,353,563.87
515-13-9	$C_{15}H_{24}$	β-Elemene	1393.042518	1391	45.828	610,068.95	45.829	809,437.76	45.823	980,486.14
547-61-5	$C_{10}H_{16}O$	(-)-trans-Pinocarveol	1133.801223	1139	27.780	1,252,533.17	27.785	1,402,806.44	27.794	1,454,975.12
5989-08-2	$C_{15}H_{24}$	α-Longipinene	1360.003681	1353	44.033	5,015,657.50	44.039	7,431,554.97	42.734	2,501,875.46
7323-15-1	$C_{12}H_{24}$	2-Isobutyl-6-methyl-1-heptene	1109.542676	1119	25.637	2,295,569.45	25.674	2,803,105.26	25.674	2,978,245.24
74410-10-9	$C_{10}H_{16}O$	Dill ether	1187.887707	1186	32.558	2,882,632.19	32.582	3,642,596.02	32.577	3,067,211.07
872-05-9	$C_{10}H_{20}$	1-Decene	1001.529008	989	17.903	2,478,064.86	17.922	4,142,849.56	17.931	3,513,768.36
1472-09-9	$C_{11}H_{22}$	Octylcyclopropane	1096.441841	1095			24.545	752,599.59	24.549	645,929.52
14912-44-8	$C_{15}H_{24}$	α-Ylangene	1373.366464	1372			44.759	10,767,214.49	44.763	8,671,653.10
20851-55-2	$C_7H_{14}O$	4-Hepten-1-ol	873.8085015	870	10.340	1,964,656.79			10.381	1,733,699.38
21129-27-1	$C_{10}H_{20}O$	Dihydroterpieol	1178.797827	1178	31.755	3,966,547.98			31.750	4,247,148.99

2243-98-3	$C_{11}H_{20}$	1-Undecyne	1075.450129	1095			23.076	22,338,105.96	23.071	19,517,188.29
2384-70-5	$C_{10}H_{18}$	dec-2-yne	1041.283224	1041	20.685	2,257,101.66	20.726	2,581,319.46		
24545-81-1	$C_{10}H_{14}O$	Umbellulone	1169.34571	1171			30.920	2,223,243.03	30.943	2,000,476.65
41432-70-6	$C_{15}H_{24}$	β-Longipinene	1395.324867	1403	46.351	3,620,314.78	46.348	2,819,583.40		
470-40-6	$C_{15}H_{24}$	Thujopsene	1418.104944	1429			47.041	1,267,492.05	47.829	631,288.55
514-51-2	$C_{15}H_{24}$	β-Patchoulene	1380.618443	1381	45.153	382,983.25	45.154	692,299.66		
5732-00-3	$C_{13}H_{16}$	2-(1,3-Butadienyl)mesitylene	1383.32413	1373	45.300	1,153,742.00			45.304	1,034,060.15

**CAS**	**Formula**	**Compound name**	**RI_theoretical**	**RI**	**Q6-1_RT**	**Q6-1_Area**	**Q6-2_RT**	**Q6-2_Area**	**Q6-3_RT**	**Q6-3_Area**
10408-15-8	$C_8H_{14}O$	6-Methyl-6-hepten-2-one	964.8042298	966	15.218	19776384.74	15.333	7345684.72	15.141	3967511.61
1204-30-4	$C_{12}H_{20}O_2$	2-Cyclohexen-1-ol, 3-methyl-6-(1-methylethyl)-, acetate	1319.639242	1303	41.83	5492358.36	41.835	6603302.05	41.84	6938714.72
14912-44-8	$C_{15}H_{24}$	α-Ylangene	1367.881465	1372	44.791	8705303.57	44.47	350744477.7	44.461	309930469.5
2384-70-5	$C_{10}H_{18}$	2-Decyne	1040.568734	1041	20.694	6925687.11	20.699	6315723.33	20.635	7813639.51
31502-14-4	$C_9H_{18}O$	trans-2-Nonen-1-ol	1178.650668	1176	31.755	2473891.2	31.778	5630756.07	31.742	6254632.52
41432-70-6	$C_{15}H_{24}$	β-Longipinene	1404.076323	1403	46.636	14621159.38	46.389	9005071.42	46.394	6875452.01

5989-08-2	$C_{15}H_{24}$	α-Longipinene	1357.426836	1353	44.423	231255800.2	44.043	6101372.09	43.893	18115914.47
74410-10-9	$C_{10}H_{16}O$	Dill ether	1187.853747	1186	32.572	3748227.86	32.586	4057725.2	32.555	4766056.3
87-44-5	$C_{15}H_{24}$	β-Caryophyllene	1420.490026	1419	47.15	5871166.22	47.151	8313640.13	47.151	11744948.64
871660-95-6	$C_{15}H_{24}$	Panaxene	1298.16655	1314	41.518	25397144.42	41.528	44773498.5	40.642	11440951.2
118-65-0	$C_{15}H_{24}$	Isocaryophyllene	1409.54033	1406			46.641	8525977.04	46.646	10890959.1
138752-23-5	$C_{15}H_{24}$	7-epi-Silphiperfol-5-ene	1352.25474	1348			43.896	26502174.29	43.612	30591130.7
15537-55-0	$C_{10}H_{18}O$	cis-Sabinene hydrate	1069.548442	1070	22.663	4145512.46			22.627	5576897.58
17699-14-8	$C_{15}H_{24}$	α-Cubebene	1349.199337	1351	43.446	4108458.54	43.437	5780102.53		
74284-57-4	$C_{15}H_{24}$	Silphinene	1352.236334	1346	43.611	23807965.87	43.621	35199495.33		
99-86-5	$C_{10}H_{16}$	α-Terpinene	1018.591026	1017	19.097	4603998.32	19.143	7426381.96		

**CAS**	**Formula**	**Name**	**RI_theoretical**	**RI**	**Q7-1_RT**	**Q7-1_Area**	**Q7-2_RT**	**Q7-2_Area**	**Q7-3_RT**	**Q7-3_Area**
10408-15-8	$C_8H_{14}O$	6-Hepten-2-one,6-methyl-	960.0064144	966	15.302	23536092.11	15.315	16471940.02	15.26	20922986.42
1128-08-1	$C_{11}H_{18}O$	Dihydrojasmone	1376.679551	1369			44.939	797611.19	44.934	781031.76
116-02-9	$C_9H_{18}O$	3,3,5-Trimethylcyclohexanol	1075.521578	1073	23.104	16621573.08	23.104	17340374.94	23.081	17521204.62

1197-06-4	$C_{10}H_{16}O$	cis-Carveol	1230.006998	1229	35.777	4120718.73	35.772	4185984.82	35.767	4036920.71
1204-30-4	$C_{12}H_{20}O_2$	2-Cyclohexen-1-ol, 3-methyl-6-(1-methylethyl)-, acetate	1319.565618	1303	41.836	7692949.78			41.834	7407639.27
138752-23-5	$C_{15}H_{24}$	cis-Sabinene hydrate	1356.488128	1348	43.842	14542779.29	43.888	5294217.2	43.887	6614669.94
14912-44-8	$C_{15}H_{24}$	alpha-ylangene	1373.29284	1372			44.765	2725801.55	44.755	3552436.31
1563-38-8	$C_{10}H_{12}O_2$	2,3-Dihydro-2,2-dimethyl-7-benzofuranol	1281.091672	1286			39.422	1657987.96	39.421	1089266.99
1569-60-4	$C_8H_{16}O$	6-Methyl-5-hepten-2-ol	989.6568313	994	17.151	3680708.83	17.161	1203856.81	17.114	1434862.94
17699-14-8	$C_{15}H_{24}$	α-Cubebene	1349.309774	1351	43.452	7189005.13	43.443	2212654.73		
17699-16-0	$C_{10}H_{18}O$	trans-Sabinene hydrate	1070.805945	1070	22.751	10996470.94	22.733	7655694.15	22.718	6990133.46
2049-94-7	$C_{11}H_{16}$	Benzene,(3-methylbutyl)-	1082.923692	1098			23.752	1056118.12	23.599	1786065.93
21653-20-3	$C_{10}H_{18}O$	Thujyl alcohol	1174.235907	1176			31.352	1323344.41	31.342	1028296.22
22469-52-9	$C_{15}H_{24}$	(+)-Cyclosativene	1370.347874	1368	44.59	1349556.59	44.595	1375956.21	44.594	1211040.73
2384-70-5	$C_{10}H_{18}$	2-Decyne	1042.212061	1041	20.75	18356434.66	20.741	17971836.15	20.712	16019041.55
31797-64-5	$C_{11}H_{22}$	tert-Pentylcyclohexane	1127.733756	1130	27.258	3002224.82	27.244	3265597.8	27.225	2851784.47
489-39-4	$C_{15}H_{24}$	(+)-Aromadendrene	1437.120555	1440	47.918	5889518.68	47.923	7772654.43	47.917	6444221.79

512-85-6	$C_{10}H_{16}O_2$	Ascaridole	1258.992302	1244			37.843	1225192.95	37.846	1021153.57
56469-37-5	$C_9H_{14}O_2$	cis-Arbusculone	1057.487854	1052			21.819	3279637.8	21.777	2701711.11
593-08-8	$C_{13}H_{26}O$	tridecan-2-one	1506.114259	1497			51.077	1069483.6	51.08	914842.66
62238-06-6	$C_{10}H_{20}$	1-Methyl-2-(1-methylpentyl)cyclopropane	945.9429121	947	14.425	1321684.96	14.379	1247755.34		
623-36-9	$C_6H_{10}O$	2-methyl-2-pentenal	828.2954058	837	8.22	16739810.46	8.302	3443939.86		
626-89-1	$C_6H_{14}O$	4-Methyl-1-pentanol	845.3413482	846	9.128	6282447.38	9.014	9075456.9		
66-25-1	$C_6H_{12}O$	Hexanal	780.5214153	800			6.379	4469792.85	6.149	2953060.25
7323-15-1	$C_{12}H_{24}$	2-isobutyl-6-methyl-1-heptene	1123.477473	1119	26.868	3684739.41	26.873	3609590.4	26.844	2637631.46
74410-00-7	$C_{10}H_{16}O$	trans-Isopiperitenol	1206.689993	1210	34.106	3350293.01	34.115	3226597.88	34.11	3068546.41
74410-10-9	$C_{10}H_{16}O$	Dill ether	1188.215984	1186			32.587	4911911.78	32.577	4859606.47
7643-59-6	$C_{10}H_{18}O$	cis-Ocimenol	1161.670817	1157	30.242	10688978.29	30.242	11788036.91	30.236	9265518.79
78-28-4	$C_7H_{15}NO_2$	Emylcamate	1096.913404	1105	24.564	1123348.3	24.578	2859880.9		
821-96-5	$C_{11}H_{22}$	(Z)-2-Undecene	1110.493548	1102	25.711	3964245.53	25.721	4105573.79		
871660-95-6	$C_{15}H_{24}$	Panaxene	1313.822934	1314	41.524	15405073.34	40.643	10274355.44	41.514	9813848.77

89-79-2	$C_{10}H_{18}O$	(-)-Isopulegol	1148.109577	1146	29.062	10850026.95	29.044	11264537.71	29.02	9905493.03

CAS	Formula	Name	RI_theoretical	RI	Q8-1_RT	Q8-1_Area	Q8-2_RT	Q8-2_Area	Q8-3_RT	Q8-3_Area
10408-15-8	$C_8H_{14}O$	6-methylhept-6-en-2-one	959.4130853	966	15.283	13821410.63	15.265	16657649.21	15.324	14637875.14
1197-06-4	$C_{10}H_{16}O$	2-Cyclohexen-1-ol,2-methyl-5-(1-methylethen yl)-, (1R,5R)-rel-	1230.076977	1229	35.767	2822635.16	35.772	2847108.24	35.777	3349319.01
1197-07-5	$C_{10}H_{16}O$	2-Cyclohexen-1-ol,2-methyl-5-(1-methylethen yl)-, (1R,5S)-rel-	1206.759972	1217	34.101	1480951.28	34.106	1876858.7	34.111	2075218.23
1204-30-4	$C_{12}H_{20}O_2$	2-Cyclohexen-1-ol, 3-methyl-6-(1-methylethyl)-, acetate	1319.565618	1303	41.831	3847190.69	41.831	4246361.69	41.836	4335863.6
123-05-7	$C_8H_{16}O$	2-ethylhexanal	952.0525978	956	14.824	779174.98	14.806	1111589.01	14.87	1021252.62
13466-78-9	$C_{10}H_{16}$	3-carene	1019.648471	1011			19.138	1852539.84	19.171	2191338.23
138752-23-5	$C_{15}H_{24}$	7-epi-Silphiperfol-5-ene	1352.25474	1348	43.828	3762048.73	43.612	17596663.42		
1472-09-9	$C_{11}H_{22}$	Octylcyclopropane	1075.978851	1095	24.527	2417944.57	23.095	28433407.49	23.113	29282974.2
14912-44-8	$C_{15}H_{24}$	α-ylangene	1373.624149	1372	44.773	7868777.48			44.774	6871687.05
17301-31-4	$C_{13}H_{28}$	3,9-dimethylundecane	1236.627012	1240	36.245	1313219.48			36.25	1181692.44

17699-14-8	$C_{15}H_{24}$	α-Cubebene	1350.064421	1351	43.493	6049858.52	43.502	4775641.44	43.438	2835277.79
2243-98-3	$C_{11}H_{20}$	1-Undecyne	1075.450129	1095	23.076	20612997.18	31.774	6028412.65		
2277-19-2	$C_9H_{16}O$	(Z)-6-Nonenal	1089.496999	1101			24.031	1909350.32	24.059	2100107.92
26532-23-0	$C_{10}H_{18}O$	Ethanol,2-(3,3-dimethylcyclohexylidene)-, (2Z)-	1219.020294	1225	36.47	651724.6	34.987	1969002.71	35.015	1860828.98
293-96-9	$C_{10}H_{20}$	Cyclodecane	1148.052977	1147			29.002	5459050.23	29.039	6404604.39
31502-14-4	$C_9H_{18}O$	trans-2-Nonen-1-ol	1173.771791	1176			31.329	2635896.55	31.311	2772615.06
33626-25-4	$C_{10}H_{18}$	trans-Pinane	989.6568313	988			17.151	2737451.06	17.188	2611015.66
3886-78-0	$C_{10}H_{16}O$	(Z)-p-Mentha-2,8-dien-1-ol	1083.581023	1102	23.645	920031.87			23.664	880637.67
41432-70-6	$C_{15}H_{24}$	β-Longipinene	1403.469211	1403	46.362	15304957.73	46.366	18191665.47	46.362	15482928.68
41678-29-9	$C_{13}H_{20}O$	Edulan I	1313.638874	1314	41.4	651001.3	41.514	12512877.41	41.409	854701.63
470-40-6	$C_{15}H_{24}$	Thujopsene	1409.258456	1429	46.633	3493984.72	46.637	3176788.85	46.95	1819744.15
475-20-7	$C_{15}H_{24}$	Longifolene	1409.258456	1405			45.581	3211378.83	46.633	3668625.45
513-23-5	$C_{10}H_{18}O$	Thujyl alcohol	1169.243831	1168	30.911	1482074.29			30.939	1381037.41
515-13-9	$C_{15}H_{24}$	β-Elemene	1392.803239	1391	45.815	3926684.31	45.82	3162102.41		

61142-79-8	$C_{11}H_{22}$	8-Methyl-1-decene	1062.074879	1056			22.14	3992702.24	22.443	3109638.14
62555-02-6	$C_{11}H_{18}O_2$	7,7-Dimethylbicyclo[2.2.1]hept-2-yl acetate	1198.086937	1181	32.371	673730.09	33.459	854691.73	32.385	781880.39
63883-69-2	$C_6H_{10}O$	(E)-2-ethylbut-2-enal	829.1756119	819	8.261	22378078.15	8.114	1890691.8		
7323-15-1	$C_{12}H_{24}$	2-isobutyl-6-methyl-1-heptene	1123.579353	1119	25.922	856722.29	25.945	1401733.78	26.877	1882891.56
74410-10-9	$C_{10}H_{16}O$	Dill ether	1188.374462	1186	32.577	3254740.6			32.601	3403974.55
7643-59-6	$C_{10}H_{18}O$	cis-Ocimenol	1161.874576	1157	30.237	5191427.05	30.26	7889224.81		
821-96-5	$C_{11}H_{22}$	(Z)-2-Undecene	1109.961512	1102	25.674	7434198.09	25.298	4262834.86		
87-44-5	$C_{15}H_{24}$	β-Caryophyllene	1428.859497	1419			47.537	2795975.16	47.152	1091025.88
871660-95-6	$C_{15}H_{24}$	Panaxene	1298.16655	1314	40.642	13060773.79	40.637	10523921.71	41.519	12232929.02

CAS	Formula	Name	RI_theoretical	RI	Q9-1_RT	Q9-1_Area	Q9-2_RT	Q9-2_Area	Q9-3_RT	Q9-3_Area
10408-15-8	$C_8H_{14}O$	6-Hepten-2-one,6-methyl-	960.3752405	966	15.325	5385569.08	15.352	4923737.15		
112-45-8	$C_{11}H_{20}O$	10-Undecenal	1284.814556	1288	39.688	1104308.44	39.692	916619.15	39.687	1005556.53
1197-06-4	$C_{10}H_{16}O$	cis-Carveol	1230.146956	1229	35.782	2732269.38	35.777	2583449.05	35.781	2131192.15
123-05-7	$C_8H_{16}O$	Hexanal, 2-ethyl-	953.5920462	956	14.87	1428138.33	14.902	1630913.79	14.805	1298479.57

13040-03-4	$C_{10}H_{16}O$	cis-Verbenol	1145.40412	1140	28.805	1211652.19	28.777	987829.5		
13151-99-0	$C_{10}H_{2}0$	Cyclooctane, 1,4-dimethyl-, cis-	1041.940554	1054	20.727	10313817.15	20.754	10892186.46	20.731	11367477.79
138752-23-5	$C_{15}H_{24}$	7-epi-Silphiperfol-5-ene	1352.659672	1348	43.846	25699060.61	43.631	54480841.8	43.634	62870999.43
15537-55-0	$C_{10}H_{18}O$	cis-Sabinene hydrate	1070.40583	1070			22.737	2770199.91	22.723	3384125.15
1563-38-8	$C_{10}H_{12}O_2$	2,3-Dihydro-2,2-dimethyl-7-benzofuranol	1281.077677	1286	39.421	4471481.69	39.421	4343544.13	39.421	3002702.33
17699-14-8	$C_{15}H_{24}$	α-Cubebene	1350.48776	1351	43.516	21663066.02	43.452	16990940.58	43.515	16433711.32
20307-84-0	$C_{15}H_{24}$	δ-Elemene	1319.547211	1338			41.84	4461749.79	41.835	5068176.93
2243-98-3	$C_{11}H_{20}$	1-Undecyne	1075.721635	1095	23.095	29163384.44	23.1	25488802.32		
22469-52-9	$C_{15}H_{24}$	Cyclosativene	1370.347874	1368	44.595	8560482.97	44.599	6309865.78	44.488	1505733.43
31502-14-4	$C_9H_{18}O$	trans-2-Nonen-1-ol	1179.227983	1176	31.793	4182413.69			31.783	3984538.81
41432-70-6	$C_{15}H_{24}$	β-Longipinene	1403.38248	1403	46.362	10998468.76	46.362	9227666.54	46.361	9302281.22
470-40-6	$C_{15}H_{24}$	Thujopsene	1409.236774	1429	46.633	1146253.05	46.633	1872012.41	46.632	1931307.87
515-13-9	$C_{15}H_{24}$	β-Elemene	1388.496227	1391	45.821	2381985.43	45.82	3941304.04	45.581	4447468.05
59742-39-1	$C_{15}H_{24}$	Cascarilladiene	1373.550525	1372	44.769	7383527.46	44.76	6310373.64		

62181-91-3	$C_{12}H_{20}O_2$	neoiso-3-thujyl acetate	1269.643107	1280			38.604	13827922.26	38.599	12513736.96
629-50-5	$C_{13}H_{28}$	Tridecane	1303.092214	1300	40.941	1067303.96	40.941	1151208.78	40.94	1079945.52
7299-42-5	C10H18O	δ-Terpineol	1162.961286	1166			30.361	14437590.81	30.356	19691258.03
74410-00-7	C10H16O	trans-Isopiperitenol	1206.885934	1210	34.12	1401724.15	34.115	1204965.01		
74410-10-9	$C_{10}H_{16}O$	Dill ether	1188.317863	1186	32.596	3220989.67	32.587	2935763.43	32.586	2858616.89
7712-79-0	C10H18O	iso-3-Thujanol	1134.027621	1136	27.8	1837398.19	27.818	1519819		
871660-95-6	$C_{15}H_{24}$	Panaxene	1314.319897	1314	41.551	56114777	41.547	45366331.25	40.665	37691187.28
89-79-2	$C_{10}H_{18}O$	(-)-Isopulegol	1148.052977	1146	29.039	5061628.59	29.039	4621206.55	29.043	5048221.41

Note: CAS, chemical formula, and compound name are provided for identification. RI_theoretical and RI represent the calculated and experimental retention indices, respectively. Q5－Q9 denote leaf samples collected from May to September. Peak areas (Area) and retention times (RT) are reported for three replicates per month (Q5－Q7 as examples in the original dataset). Only compounds detected in at least two months are included in this summary. This table serves as the supplementary dataset to Figures 4 and 7, supporting tentative and semi-quantitative analysis of VOCs in the study.
